# DYW domain structures imply an unusual regulation principle in plant organellar RNA editing catalysis

**DOI:** 10.1038/s41929-021-00633-x

**Published:** 2021-06-21

**Authors:** Mizuki Takenaka, Sachi Takenaka, Tatjana Barthel, Brody Frink, Sascha Haag, Daniil Verbitskiy, Bastian Oldenkott, Mareike Schallenberg-Rüdinger, Christian G. Feiler, Manfred S. Weiss, Gottfried J. Palm, Gert Weber

**Affiliations:** 1 Department of Botany, Graduate School of Science, Kyoto University, Kyoto, Japan; 2 University of Greifswald, Molecular Structural Biology, Greifswald, Germany; 3 Molekulare Botanik, Universität Ulm, Ulm, Germany; 4 Institut für Zelluläre und Molekulare Botanik, Abteilung Molekulare Evolution, University of Bonn, Bonn, Germany; 5 Helmholtz-Zentrum Berlin für Materialien und Energie, Macromolecular Crystallography, Berlin, Germany; 6 Present address: Helmholtz-Zentrum Berlin für Materialien und Energie, Macromolecular Crystallography, Berlin, Germany.; 7 These authors contributed equally: Sachi Takenaka, Tatjana Barthel

## Abstract

RNA editosomes selectively deaminate cytidines to uridines in plant organellar transcripts–mostly to restore protein functionality and consequently facilitate mitochondrial and chloroplast function. The RNA editosomal pentatricopeptide repeat proteins serve target RNA recognition, whereas the intensively studied DYW domain elicits catalysis. Here we present structures and functional data of a DYW domain in an inactive ground state and activated. DYW domains harbour a cytidine deaminase fold and a C-terminal DYW motif, with catalytic and structural zinc atoms, respectively. A conserved gating domain within the deaminase fold regulates the active site sterically and mechanistically in a process that we termed gated zinc shutter. Based on the structures, an autoinhibited ground state and its activation are cross-validated by RNA editing assays and differential scanning fluorimetry. We anticipate that, in vivo, the framework of an active plant RNA editosome triggers the release of DYW autoinhibition to ensure a controlled and coordinated cytidine deamination playing a key role in mitochondrial and chloroplast homeostasis.

Plant RNA editing specifically converts several hundreds of cytidines to uridines in mitochondrial and chloroplast transcripts^1–4^. The RNA editing activity has to be stringently suppressed in the cytosol and stimulated only in the organelles at the target site, as no C-to-U RNA editing has been described in nuclear transcripts in plants^5,6^. Nuclear-encoded pentatricopeptide repeat (PPR) proteins with a C-terminal DYW domain have been characterized as site-specific factors for C-to-U RNA editing in plant mitochondria and plastids^7,8^. RNA substrate recognition is conferred by the PPR tract, whereas the exact role of the DYW domain, which can be also recruited to an editing site in trans, has not been clarified^9–17^. The DYW domain, which was named by the highly conserved last three amino acids, aspartate, tyrosine and tryptophan, has been proposed as the best candidate to elicit deamination employing a HxE(x)_*n*_CxxC zinc ion binding signature^18,19^. Indeed, DYW domains from DYW1 and ELI1 demonstrated zinc ion binding capacity, and recent orthogonal *E.coli* as well as in vitro experiments with a single DYW containing PPR protein strongly support their function as catalytic entities within the RNA editosome^20–23^. Apart from PPR proteins and DYW domains, several other factors (for example MORF or ORRM proteins) were shown to be part of RNA editosomes^6,10,24,25^. Until now, only MORF proteins and PPR repeats bound to the target RNA are structurally characterized^13,26–28^. As DYW domains share only low sequence conservation with known deaminase structures (from 5 to 19% residue identities), modelling attempts have been conducted, albeit with a limited reliability^18,20,21,29^. Finally, missing structural information has left the exact mechanistic function, regulation and catalytic properties of DYW domains within the RNA editosome open.

Herein we describe structures of a DYW domain and find that, apart from a cytidine deaminase fold, DYW domains contain a characteristic DYW motif, stabilized by a zinc atom, as well as a gating domain that controls zinc-mediated catalysis sterically and catalytically. The catalytic regulation hallmarks an unusual protein regulation principle where, upon activation, a major movement of the gating domain alters the coordination around the catalytic zinc atom while in the inactive state, the zinc is inhibited by its coordination setting. We employed in vivo RNA editing assays to map the potential RNA path on the DYW domain and identify key residues required for regulation and catalysis to occur. Finally, RNA in vitro editing and thermal shift assays consolidate the structural data and confirm a tetrahydrouridine or nucleotide triphosphate-triggered activation mirroring the two different conformational states. Beyond the identification of an unusual principle in metalloenzyme regulation, our results reveal key mechanisms in plant organellar RNA editing catalysis, its autoinhibition and have far-reaching implications for mitochondrial and chloroplast homeostasis.

## Results

### Crystal structure of the *Arabidopsis thaliana* OTP86^DYW^

Here we report crystal structures of the DYW domain of an *Arabidopsis thaliana* (*A. thaliana*) plastid RNA editing factor, OTP86, as the outcome of a solubility and crystallization screening of over 100 different DYW domain constructs from 30 PPR proteins. OTP86 was characterized as a site-specific factor for an editing site in *rps14* transcripts^30^. The protein consists of 20 N-terminal PPR repeats, E1 and E2 motifs, which are predicted to have a PPR- or tetratricopeptide-repeat-like (TPR-like) fold and a C-terminal DYW domain^31^.

To initially assess whether the OTP86 DYW domain (OTP86^DYW^) is an active editing factor, we conducted in vivo orthogonal RNA editing assays in *Escherichia coli* (*E. coli*) and in vitro assays with purified proteins. Both methods verified the cytidine deaminase activity of the OTP86 DYW domain when fused with the PPR tract of the moss *Physcomitrium* PPR56 protein (Supplementary Fig. 1a–c)^22,23^. When the catalytically important E894 of OTP86^DYW^ was replaced by an alanine, editing was abolished.

We then set out to pioneer the structural characterization of DYW domains exemplified by OTP86^DYW^. Several years of crystallization attempts were severely hampered by the very limited amounts of soluble OTP86^DYW^ (residues G826 to W960), which migrates at a molecular weight of about 15 kDa in size-exclusion chromatography, indicating a monomeric state (Supplementary Fig. 2a). Finally, we obtained crystals of OTP86^DYW^ belonging to space group C2 and diffracting to a resolution of 2.5 Å (Supplementary Fig. 3a and Supplementary Table 1). The structure was solved by single-wavelength anomalous dispersion (SAD) phasing harnessing four zinc atoms (see Methods, Supplementary Table 1 and Supplementary Fig. 3b, c for details).

**Fig. 1:**
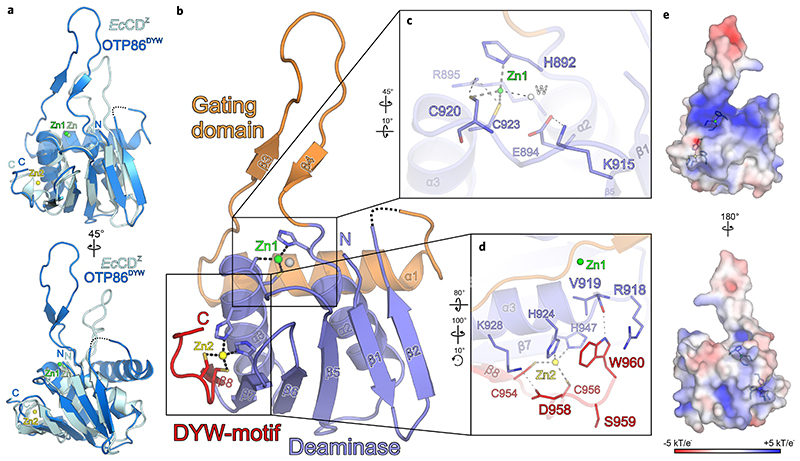
Crystal structure of the *A. thaliana* OTP86 DYW domain. **a**, Superimposition of OTP86^DYW^ (marine) with *E. coli* cytidine deaminase (*Ec*CD^Z^, cyan) bound to the inhibitor zebularine (not shown) (PDB-ID: 1CTU; ref. ^32^). The consensus deaminase zinc ions are shown as green (OTP86^DYW^, Zn1) and light-green (*Ec*CD^Z^, Zn) spheres, a zinc ion partially coordinated by the DYW motif is shown in yellow (Zn2). **b**, The OTP86^DYW^ structure defines a paradigmatic organization for DYW domains. The cytidine deaminase domain (slate) coordinates a zinc ion (Zn1, green) three-fold with H892, C920 and C923, the fourth position is occupied by a water molecule (W, white sphere). The deaminase domain is interrupted by a gating domain (orange) and terminates with a DYW motif (red), partially coordinating a second zinc ion (Zn2, yellow). **c**, A close-up view on the cytidine deaminase active site, with catalytically relevant residues shown as sticks. **d**, A close-up view of the DYW motif and the flanking β-strand 7 as well as α-helix 3. **e**, Electrostatic surface potentials as indicated by the colour scale bar (bottom), obtained by APBS version 1.5 and plotted on the surface of OTP86^DYW^. Residues involved in zinc coordination are shown as sticks; zinc atoms are as in **b**. Rotation symbols indicate the views relative to **b**. Interacting residues are shown as sticks and coloured by atom type. Blue, nitrogen; red, oxygen; yellow, sulfur; carbons take the colour of the respective molecule. Dashed lines represent hydrogen bonds, whereas thick grey dashed lines indicate zinc coordination. Dashed lines in the ribbon plots represent residues 842-844 not clearly defined by electron density.

**Fig. 2:**
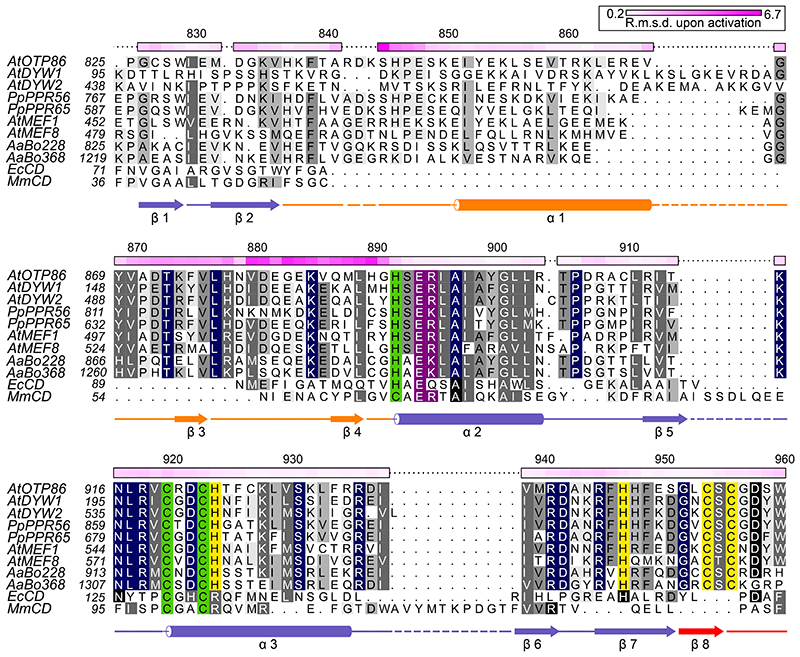
Structure-based sequence alignment of OTP86^DYW^. The alignment compares OTP86^DYW^ with plant organellar DYW domains, hornwort putative U-to-C aminases^53,72^, C-to-U deaminases from *E. coli* (*Ec*CD, PDB-ID: 1AF2; ref. ^43^), and *Mus musculus* (*Mm*CD, PDB-ID: 2FR6; ref. ^44^). The alignment was prepared by Chimera employing Clustal Omega and shaded with ALSCRIPT^73,74^. Proteins are identified on the left of the aligned sequences with residues numbered (see Supplementary Table 2). Higher conservation is indicated by a darker background. Full conservation within DYW domains is indicated by dark blue background shading. Single-residue C**α** r.m.s.d. values (calculated with CCP4i) between OTP86^DYW^ and OTP86^DYW*★*^ (or structural changes upon activation) are displayed on the top of each alignment block as a colour gradient from white to pink (prepared with Python Matplotlib) and are quantified in the top-right box. The overall r.m.s.d. between the two structures was 1.99 Å, the gating domain residues 870–891 (β-finger) amounted to 2.98 Å. The numbering on top of each block refers to *A. thaliana* OTP86. Below the alignment blocks, secondary structure elements (**α**–**α**-helix, β-β-sheet) of OTP86^DYW^ are shown in slate for the deaminase domain, orange for the gating domain and red for the DYW motif. A dashed line or no line indicates residues missing from the structure or the expression construct, respectively. Green shading indicates residues coordinating the catalytic Zn1 ion; yellow shading indicates residues coordinating the Zn2 atom within the DYW motif and strand β7/helix **α3**; and purple shading indicates residues relevant for catalysis according to past studies. *At*, *Arabidopsis thaliana*; *Pp*, *Physcomitrium patens*; *Aa*, *Anthoceros agrestis*.

The fold of OTP86^DYW^ is highly similar to cytidine deaminases but has prominent additional features (Fig. 1a–e). A comparison of OTP86^DYW^ with *E. coli* cytidine deaminase (PDB ID: 1CTU; ref. ^32^) reveals an overall similarity (r.m.s.d. = 2.4 Å for 72 of 132 residues superimposed) to the typical core deaminase fold comprising five β-strands flanked by two α-helices^32^. The region previously termed PG box covers the first two β-strands of the deaminase domain^21,29,33^. Remarkably, the deaminase fold of OTP86^DYW^ is interrupted by an insertion of about 55 residues that bridge β-strand 2 and α-helix 2 (Figs. 1b and 2). The insertion is composed of an amphipathic α-helix that runs across one face of the entire structure contacting both α-helices of the deaminase fold with conserved hydrophobic residues (Supplementary Fig. 3d and Fig. 2) and re-enters the deaminase fold via a highly conserved β-finger at α-helix 2, which in turn harbours the HxE(x)_*n*_CxxC motif, crucial to catalysis and substrate binding^29^ (Figs. 1b, c and 2). This motif has a high similarity to the cytidine deaminase signature HxE(x)_*n*_PCxxC and contains a catalytically important glutamate residue (E894 in OTP86), only the proline is not conserved in DYW domains (Fig. 2)^18^. Contrasting the large inserted domain of OTP86^DYW^, *E. coli* cytidine deaminase only contains a smaller loop which instead points away from the active site permitting nucleotide entry (Fig. 1a). We conclude that the OTP86^DYW^ active site seems to have limited accessibility for sub-strate cytidines, which is conferred by an insertion (H837-G891), and we thus term this insertion gating domain.

The gating domain is shared by DYW domains of all land plant clades (Figs. 1b and 2, and Supplementary Fig. 4), suggesting a conserved C-to-U RNA editing mechanism. DYW1 and DYW2 of *Arabidopsis thaliana*, however, show a less conserved N-terminus of the gating domain (Fig. 2)^12,15,17^, but interaction with E+ type PPR proteins that carry C-terminally truncated DYW domains may restore the functionality of these deviant gating domains again.

The arrangement of the active-site zinc ion coordination corroborates past in vivo studies in which mutants of the HxE(x)_*n*_CxxC zinc ion binding signature showed no editing activity (Fig. 1c)^20,23,34^. The highly conserved OTP86^DYW^ E894, which was previously hypothesized to transfer a proton from the substrate water molecule to ammonia during catalysis (see Supplementary Note 1), was shown to be essential for in vivo editing^20,23,35–37^. Notably, R895 hydrogen bonds to C920 and compensates the negative charge of the active site together with the dipole moment of helix α3 in a similar fashion as observed for *Bacillus subtilis* cytidine deaminase^38^. As a third hallmark of the DYW domain structure, the nine C-terminal residues form a structural element that we termed a DYW motif, which is represented by an additional β-strand and a short loop. In OTP86^DYW^, the motif terminates with the DSW sequence and provides two ligands (C954, C956) for a second zinc ion (Zn2); two more ligands are part of the deaminase domain (H924 and H947), indicating that Zn2 only has a structural role within DYW domains (Figs. 1d and 2). We employed X-ray fluorescence spectroscopy on OTP86^DYW^ crystals to assess whether divalent metals other than zinc were present in our structure. A comparison of the spectrum taken from solvent area in the sample loop with an OTP86^DYW^ crystal confirmed zinc as the only relevant signal detected between calcium and copper (Supplementary Fig. 2c, d). Other ions do not fit in the observed coordination geometries and electron densities. Any alteration of the residues involved in the coordination of Zn2 abolished RNA editing in vivo, which is probably due to destabilization of the entire motif^34^. The tryptophan at position 960 in OTP86^DYW^ flanks Zn2, is highly conserved in DYW domains and was shown to be essential for deaminase function in vivo for DYW1 and *Pp*PPR65^20,23^. Notably, the surface charge distribution of OTP86^DYW^ reveals a region of positively charged residues spanning across the active site and passing in between the base of the gating domain’s β-finger and the DYW motif. As RNA bases around the editing site are not conserved, this probably represents the path of the negatively charged RNA backbone, which is placed for catalysis by the PPR tract after or concomitant to activation of the DYW domain (Fig. 1e).

### Crystal structure of an activated *A. thaliana* OTP86^DYW^

**Fig. 3:**
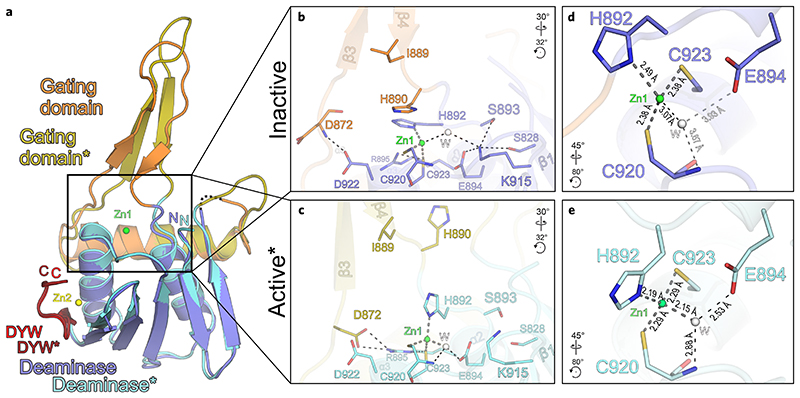
The DYW gating domain regulates cytidine deamination catalysis. **a**, Superimposition of inactive OTP86^DYW^ (colouring and dashed lines are as in Fig. 1b) and activated OTP86^DYW*★*^ (deaminase domain, cyan; gating domain, ochre; DYW motif, dark red). Zn1 (green) and Zn2 (yellow) of OTP86^DYW^ are shown. **b**, A close-up view of the OTP86^DYW^ active site in the inhibited state. **c**, A close-up view of the activated OTP86^DYW*★*^ active site in a catalytically competent conformation. **d**, A close-up view of the Zn1-coordination environment of the OTP86^DYW^ active site in its catalytically inhibited state. **e**, A close-up view of the Zn1-coordination environment of the OTP86^DYW^ active site in its catalytically active state. Distances within the vicinity of Zn1 are given in Å. Rotation symbols indicate the views relative to **a**. Interacting residues are shown as sticks and coloured by atom type. Water molecules (W) shown as white spheres. Carbon — as for the respective molecule; nitrogen, blue; oxygen, red; sulfur, yellow. Dashed lines represent hydrogen bonds, thick grey dashed lines indicate zinc coordination.

As crystal soaking experiments with substrate, product or different short RNA trinucleotides were unsuccessful, we attempted co-crystallization of OTP86^DYW^ with the well-characterized deaminase inhibitor tetrahydrouridine (THU)^30^. Along this approach, we observed several new crystallization conditions that indicated a different crystallization behaviour due to the presence of THU. Finally, we obtained crystals of OTP86^DYW^ with space group *P*2_1_2_1_2, which diffracted to a resolution of 1.65 Å (Supplementary Table 1). When employing the coordinates of OTP86^DYW^, structure solution by molecular replacement failed; however, four copies of a truncated model missing the gating domain were successfully placed in the asymmetric unit (see Methods, Supplementary Table 1 and Supplementary Fig. 5). Explaining the failed molecular replacement, OTP86^DYW^ had clearly changed its conformation substantially in the presence of THU towards an activated (OTP86^DYW*★*^) state (Fig. 3a, Supplementary Fig. 6 and Supplementary Video 1). The conformational change mainly involved the β-hairpin of the gating domain (which now adopts an extended β-strand conformation), and its connection to α-helix 1 (gating domain) and α-helix 2 (deaminase domain). It is widely accepted that conformational switches of β-fingers may take part in the regulation of macromolecular complexes as observed for the RNAse H domain in the spliceosomal Prp8 protein^39,40^. The conformational change has a marked effect on the active site architecture, in particular zinc coordination. The inactive structure zinc coordination is maintained by coordinating H892, C920, C923 and a more distant water molecule, whereas the catalytically important E894 is ionically bonded to K915 (Fig. 3b); K915 also hydrogen bonds to S828 and S893. In this configuration, the ion pair will reduce the basic character of E894 and hinder the required deprotonation of the deaminating water, which is not productively coordinated by Zn1 and also not contacted by E894. We reason that beyond the steric inhibition through the gating domain, K915 has to be released before efficient catalysis can occur. This notion is corroborated by the activated OTP86^DYW*★*^ (Fig. 3d), in which K915 points away from E894. Although E894 is conserved in all deaminases, K915 is restricted to DYW domains (Figs. 1c and 2)^38^. The conformational changes upon activation involve several larger backbone torsion angle movements of the gating domain’s β-hairpin.

The direct effects of the gating domain’s conformational change on OTP86^DYW^ catalytic activation via Zn1 coordination are evident from the detailed structural comparison of the zinc coordination and E894 (Fig. 3d,e, and Supplementary Videos 1 and 2). Remarkably, the conformational change of H892 from the main chain dihedral angles angles *ϕ*/*ψ* = 58°/42° (inactive) to *ϕ*/*ψ* = −74°/152° (active) and a concomitant repositioning of its side chain elicits a pervasive impact on the Zn1 coordination geometry (Fig. 3b, c). When superimposing residues C920–C923 of both structures, activation moves the coordinating nitrogen of H892 by 2 Å, with a concomitant rotation around the Zn1 coordination sphere by about 35° that harnesses the zinc ligands C920 and C923 as a rotation axis; C920, C923 and Zn1 remain largely unaffected during activation. The restructuring of the active site reduces the zinc-water/water-E894 distances from 3.07 Å/3.93 Å (inactive conformation) to 2.15 Å/2.53 Å, thereby activating the mechanistically important water molecule (Supplementary Video 2). The altered H892 positioning permits the remotely located water molecule to be attracted to Zn1 as a fourth coordination ligand poised for the deamination reaction, and the water molecule is now situated in close vicinity to E894 as well as the C920 amide (Fig. 3d,e and Supplementary Figs. 5 and 6). In OTP86^DYW*★*^, distances and angles of the zinc ligands are in agreement with a catalytically competent reaction centre^41,42^. Furthermore, the strand length of the β-finger is extended upon activation, still maintaining the original backbone hydrogen bonding residue pairs of the inactive OTP86^DYW^. The side chain of H890, which shields the active site as a counterpart relative to the zinc coordination sphere (formed by H892, C920 and C923) in the inactive OTP86^DYW^, is repositioned far away from the active site by about 13 Å in OTP86^DYW*★*^ (compare Fig. 3b,c, Supplementary Video 1). Unexpectedly, THU could not be located in the electron density, which implies a crucial role in triggering activation but not as tightly bound inhibitor. We are not aware of a comparable mechanism and thus coin this catalytic activation mechanism of DYW domains, and probably other metalloenzymes, gated zinc shutter.

### Structural comparison of the OTP86 DYW domain to other cytidine deaminases

**Fig. 4:**
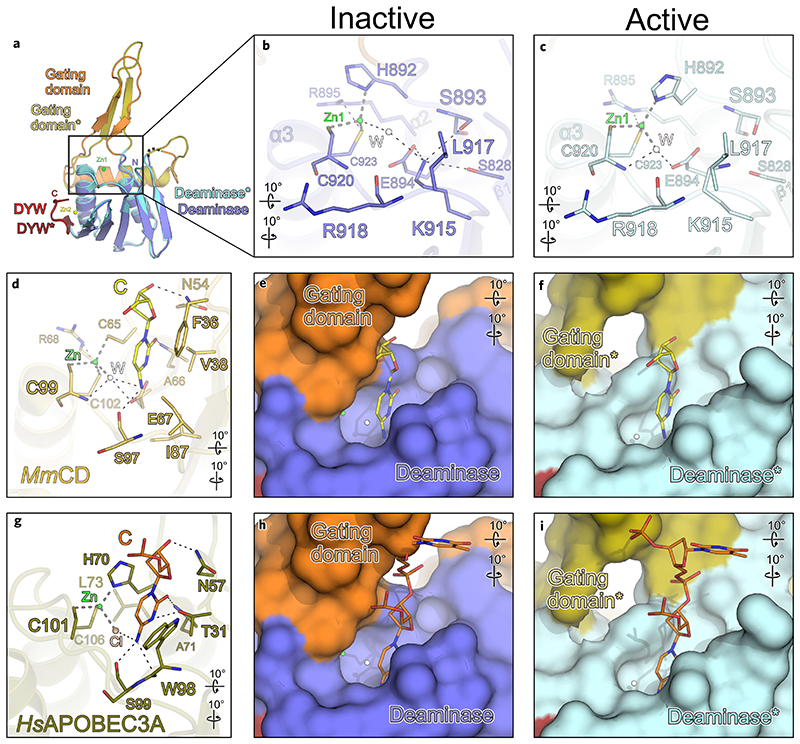
The OTP86^DYW^ active site is sterically regulated by the gating domain. **a**, Superimposition of inactive OTP86^DYW^ and activated OTP86^DYW*★*^ (as in Fig. 3a). **b**, A close-up-view of the OTP86^DYW^ active site in the inhibited state, depicting catalytic and potential RNA binding residues. **c**, A close-up-view of the OTP86^DYW*★*^ active site. **d**, *M. musculus* cytidine deaminase (*Mm*CD; light orange) in complex with cytidine (yellow), coordinated zinc (green sphere) and an activated water molecule (white sphere) (PDB-ID: 2FR6; ref. ^44^) **e**,**f**, A surface display of the active site cavity of OTP86^DYW^ (**e**) and OTP86^DYW*★*^ (**f**) with the superimposed cytidine from **d**. **g**, Human APOBEC3A (dark ochre) with bound DNA (orange, only the active site cytidine is shown for clarity), coordinated Cl (light pink) (PDB-ID: 5KEG; ref. ^75^). **h**,**i**, A surface display of the active site cavity of OTP86^DYW^ (**h**) and of OTP86^DYW*★*^ (**i**) with the superimposed DNA from **g**. The cytidine deaminase structure of *M. musculus* and human APOBEC3A were superimposed employing only the zinc-coordinating residues and the equivalents of OTP86^DYW^ E894. The colouring and dashes are as in Fig. 3. Rotation symbols indicate the views relative to **a**.

A comparison of OTP86^DYW^ with known ligand-bound deaminase domains confirms the presence of a complete active site for catalysis and fortifies the notion of a steric autoregulatory mechanism for DYW domains (Fig. 4a–i). When comparing OTP86^DYW^ with cytidine deaminase from mouse bound to cytidine (*Mm*CD), or human APOBEC3A in complex with a short DNA (*Hs*APOBEC3A), nearly all of the residues required for nucleotide binding are present in OTP86^DYW^ and located at corresponding positions (Fig. 3b–d,g)^43,44^. For example, all atoms of residues coordinating Zn1 and E894, as well as the backbone R918 carbonyl, C920 and S893 amides (contacting the respective base in *Mm*CD and HsAPOBEC3A) of OTP86, superimpose to their mouse and human equivalents with r.m.s.d. values of 1.0 and 0.9 Å, respectively. The backbone carbonyl oxygen of R918 or the backbone amide of C920 are within hydrogen bonding distance to the amine of the base or the activated water molecule, respectively (compare Fig. 4b,c with Fig. 4d,g). Likewise, the backbone carbonyl oxygen of S893 (OTP86) may contact the keto group of the bound cytidine as for A66 in *Mm*CD or A71 in *Hs*APOBEC3A. L917 of OTP86^DYW^ (Fig. 4b,c) has equivalent residues (I87 in *Mm*CD or W98 in human APOBEC3A) that stack on the edited base (Fig. 4b,d,g). H70 in *Hs*APOBEC3A adopts a similar side chain conformation as H892, however, only in OTP86^DYW*★*^, implying a role in base stacking upon activation (compare Fig. 4b,c,g). The OTP86-equivalent residue for *Mm*CD N54 or *Hs*APOBEC3A N57, both of which contact the sugar 3′ oxygen, could not be identified. This residue may also be part of a region preceding the OTP86^DYW^ deaminase fold (or PG box), which is missing in our structure. Hence we conclude that the OTP86^DYW^ active site and the positioning of the base targeted for deamination is nearly identical to other cytidine deaminases. The absence of the region preceding the PG box from our crystallization constructs may have impeded our attempts to obtain structures of OTP86^DYW^ bound to substrate-related molecules.

A superimposition of bound nucleotides of known deaminase structures has further implications for the OTP86 activation mechanism. To investigate the nucleotide binding mode of OTP86^DYW^, we superimposed active site residues of *Mm*CD and *Hs*APOBEC3A onto OTP86^DYW^ in its activated and inactive states and compared the substrate positions (Fig. 4e,f,h,i). For example, the cytidine bound to *Mm*CD causes steric clashes with the β-finger of the OTP86^DYW^ gating domain when positioned in the active site of OTP86^DYW^. Due to the conformational change of the gating domain upon OTP86^DYW^ activation, this inhibition is released (compare Fig. 4e,f). When comparing OTP86 structures with DNA-bound human APOBEC3A, the +1 nucleotide (3′ of the active site) causes steric clashes with the gating domain only in the inactive OTP86^DYW^ conformation, but not in OTP86^DYW*★*^. In conclusion, several superimposed substrate nucleotides suggest a steric inhibition by the OTP86 gating domain and probably other DYW deaminases as key residues (1) for nucleotide positioning and (2) participating in the conformational changes show a high degree of conservation (Fig. 2). These observations consolidate the notion of an autoinhibited ground state of OTP86^DYW^, which is paradigmatic for all PPR proteins with a DYW domain.

### In vivo RNA editing assays with OTP86^DYW^ and variants

**Fig. 5:**
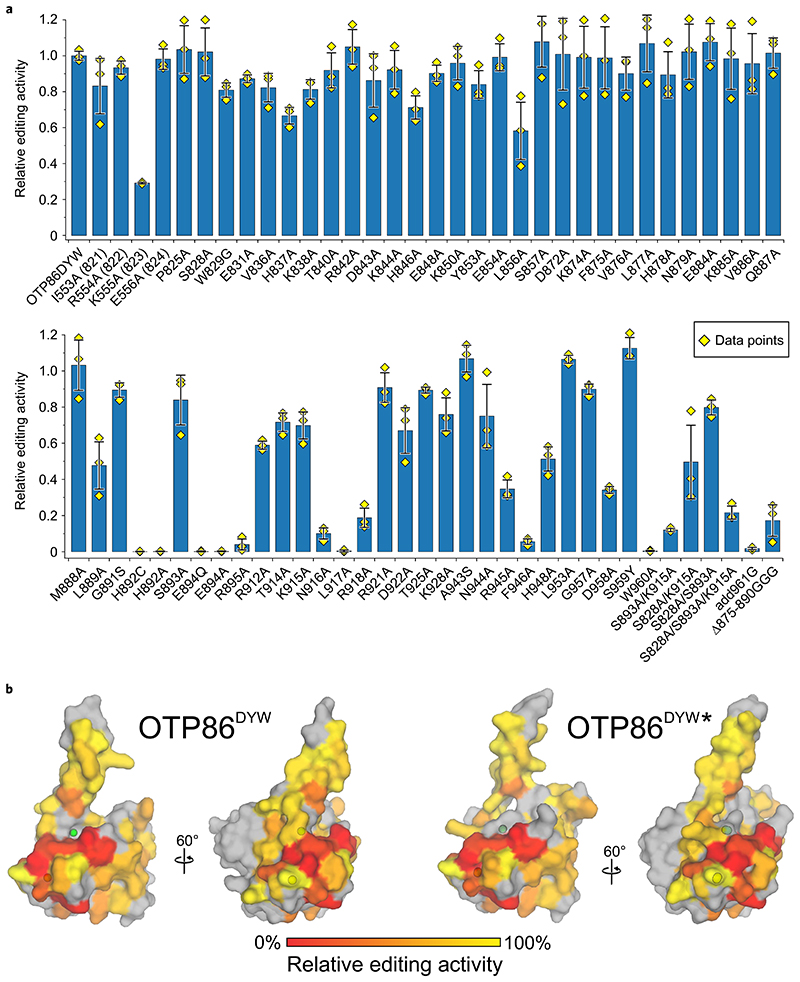
Orthogonal in vivo RNA editing validates the OTP86^DYW^ domain structure and activation. **a**, The C-to-U editing activities at the nad4eU272SL site in *E. coli* expressed with the PPR56^PPRE1E2^-OTP86^DYW^ fusion protein (OTP86DYW), or its mutants, are plotted. The activities of mutants are relative to that of PPR56^PPRE1E2^-OTP86^DYW^ (82.4 ± 2.1% edited). The bars represent the mean values, with each mutant protein ±s.d. based on three independent experiments (shown as yellow diamonds). The soluble protein expression of each mutated construct in *E. coli* was verified by western blot analysis (Supplementary Fig. 7). **b**, The activities of OTP86^DYW^ mutants shown in **a** plotted on the surface of the inactive OTP86^DYW^ and the activated OTP86^DYW*★*^ structure as a heatmap (activity is scaled in the bar on the bottom), with untested residues shown in grey.

To cross-validate the structural data and also probe the DYW domain surface, we conducted orthogonal in vivo RNA editing assays in *E. coli* employing PPR56^PPRE1E2^–OTP86^DYW^ mutants (Fig. 5)^23^. The solubility of the mutants was assessed by a western blot employing the soluble fraction of the respective cell lysates (Supplementary Fig. 7). To this end, the reduced activities of for example, L856, R912, T914, D922 can be explained by the very limited solubility of the respective fusion proteins. By contrast, K555A (PPR56 numbering, corresponding to position K823 in OTP86) is soluble and the mutant has a dramatically reduced editing activity. In OTP86, the equivalent lysine is located directly before the PG box at position 823 and may contact the sugar of the edited nucleotide or the acidic phosphate backbone, for example as N54 in *Mm*CD (see Fig. 4d). L889 directly precedes the active site as part of the β-finger, changes its position upon activation and may contact the RNA substrate remotely from the edited base, probably explaining its reduced in vivo RNA editing activity (Fig. 3b,c). H892 is a key regulatory residue as it alters its zinc coordination position upon activation, which poises the active site for the reaction. An alanine at this position is inactive as it is not suitable as a zinc coordination ligand. A cysteine may coordinate the zinc; however, H892C is also inactive. We reason that either the cysteine side chain does not provide the necessary flexibility to undergo a dramatic repositioning as that of histidine does. Alternatively, cysteine is a strong coordination ligand of zinc compared with histidine and may thus reduce zinc reactivity. S828 and S893 apparently play an important inhibitory role when contacting K915 and tethering it to the catalytically important E894 (Fig. 3b and Supplementary Note 3).

Mutants of the catalytic residue E894 retain their solubility upon mutation to alanine or the structurally analogous uncharged glutamine; however, both mutants are inactive, which consolidates their important role in deamination catalysis^22,23,35^. Albeit soluble to a low degree (compare with D922), the R895A mutant is probably inactive due to a structural destabilization of the active site. Alternatively, R895 may be crucial to catalysis according to the previously described zinc charge compensation^38^. Interestingly, the hydrogen bond donor of R895 changes from the terminal N^η^ to the weaker bridging N^ε^ during activation. In the active conformation, R895 hydrogen bonds to D872 (Fig. 3b,c). This stabilizing effect is missing in the R895A mutant, which could possibly be an explanation for high conservation and R895A inactivity. Conversely, D922 stabilizes the inactive state of OTP86^DYW^; however, an alanine mutant has reduced activity. We can explain this effect as a result of the inactive ground state (destabilized through D922A) very likely being required for repetitive reactions elicited by a single DYW domain. Mutations of R945, D958 and W960 to alanine show reduced activity, which can be structurally explained by destabilizing effects on OTP86^DYW^. W960 is tightly embedded in the DYW motif and stabilizes it as it stacks on top of zinc-coordinating H924 beneath highly conserved R918 and maintains a hydrogen bond to the backbone oxygen of V919.

Likewise, D958 consolidates the DYW motif by formation of a hydrogen bond to highly conserved K928, which explains the impaired function of a respective aspartate mutant in in vivo editing assays with DYW1^20^ and finally our catalytically impaired D958A mutant. S959 in OTP86 (or tyrosine in most DYW domains) points into the solvent, thus, mutation of the corresponding tyrosine to alanine has no effect on DYW1 in vivo activity^20^ and the reverse mutation has no effect on OTP86 activity in this work (Fig. 5a); however, an phenylalanine to alanine mutation at this position in *Physcomitrium* PPR65 showed a severe negative impact on editing^23^. Our structure may help to interpret these past in vivo mutagenesis studies in several ways. Most likely, an impaired stability of the DYW motif as pictured above triggers a destabilization of the active-site Zn1 as they are directly linked via helix α3, which provides residues coordinating Zn1 and Zn2 (Figs. 1b and 2, and Supplementary Fig. 3b,c). In this context, the DYW motif may also play a role in regulation, for example, the release of the gating domain, repositioning of K915 or binding nucleotides adjacent to the editing site. Finally, we replaced the gating domain’s β-finger residues 875–890 with three glycine residues (Δ875–890GGG). The removal of the entire β-finger markedly reduces the editing activity, which implies its important functional role—probably during activation, dimerization or RNA binding—conferred by this region in DYW domains. Although size-exclusion chromatography of isolated OTP86^DYW^ and in the presence of activators (Supplementary Fig. 2a,b) did not indicate dimer formation, prominent protein–protein contacts within the crystal lattice may be physiologically relevant (Supplementary Fig. 8 and Supplementary Note 3)^45^.

### Validation of the OTP86^DYW^ activation mechanism in vitro

**Fig. 6:**
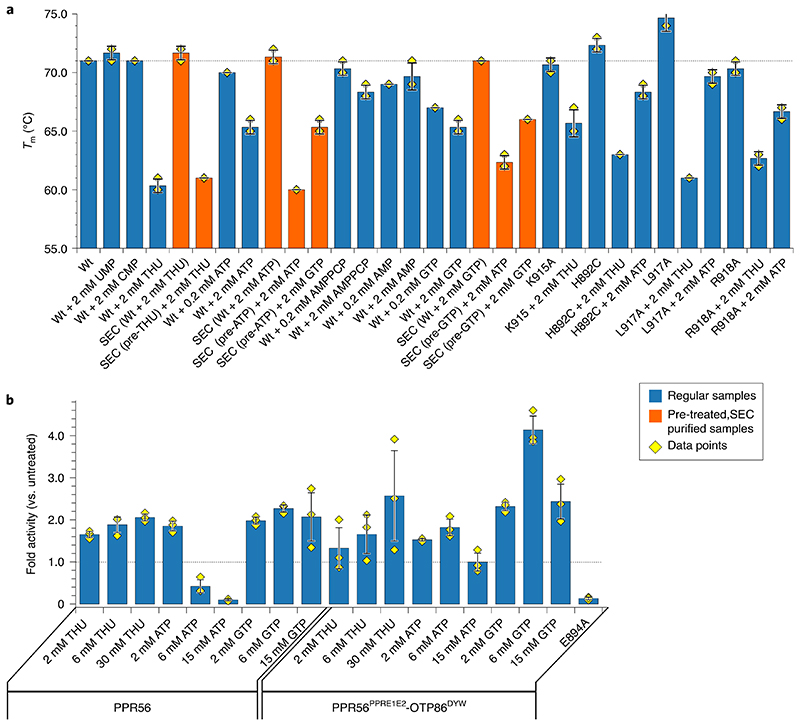
in vitro editing and thermal shift assays validate the activation principle of OTP86^DYW^. **a**, Melting points of OTP86^DYW^ and selected variants in the presence of substrate, product and activators. Thermal shift assays were performed in triplicates. Curves were subjected to sigmoidal fitting; error bars indicate the s.d. of the three measurements (shown as yellow diamonds); regular samples are shown as blue bars, whereas SEC-related experiments are shown as orange bars; ‘pre’ indicates a sample pre-incubated with the respective activator and purified by SEC. **b**, C-to-U conversion for in vitro reactions with recombinant PPR56 or PPR56^PPRE1E2^-OTP86^DYW^ with the addition of THU, ATP or GTP are displayed in a bar plot. In vitro editing with recombinant PPR56^PPRE1E2^-OTP86^DYW-E894A^ mutant protein showed no editing activity. Bars indicate the mean value ±s.d. based on three independent experiments (shown as yellow diamonds). A grey dashed line indicates a *T*_m_ of 71°C for wild type protein (WT in **a**) or the activity of the untreated proteins (in **b**).

We next set out to cross-validate these distinct structural changes of isolated OTP86^DYW^ in solution. The very low amounts of available OTP86^DYW^ led us to conduct differential scanning fluorimetry (DSF). In a typical DSF experiment, an increase of the protein’s melting point (*T*_m_) upon ligand binding is observed^46^. The substrate (CMP), product (UMP) and a K915A mutation do not have an effect on the overall high *T*_m_ of OTP86^DYW^, which is about 71–2 °C in each case (Fig. 6a)^47^. These results imply a limited accessibility of the active site due to steric inhibition, consistent with the structures and corroborated by mutants. The well-characterized-transition-state analogue THU lowers the *T*_m_ of OTP86^DYW^ to 60 °C, corresponding to the structural changes we observed following THU co-crystallization. We reason that THU, a potent cytidine deaminase inhibitor, outcompetes the gating domain from the active site, for example, by releasing H890, opening the protein up for substrate access and thereby destabilizing OTP86^DYW^ markedly^47,48^. Interestingly, this effect is less severe with the K915A mutation, implying a functional role of K915A during activation but not catalysis. Next we asked whether the effect of THU is reversible. A THU-pre-treated OTP86^DYW^ sample was therefore subjected to size-exclusion chromatography to remove THU. Indeed, repurified OTP86^DYW^ resembled the inactive state with a *T*_m_ of 72 °C despite THU exposure beforehand. The active state could be restored by the addition of 2 mM THU, resulting in a *T*_m_ of 61 °C. We conclude that DYW domains have an inhibited ground state that is restored after the activation and an editing event.

ATP was reported to activate in vitro RNA editing reactions with a recombinant *Physcomitrium* PPR65 protein as well as with plant organellar lysates^22,49,50^; we thus also tested ATP and a concentration of 2 mM was required to drop the *T*_m_ to 65 °C. A very similar picture was obtained by addition of GTP, where 2 mM GTP reduced the *T*_m_ to 65.3 °C. Like THU, the activators ATP and GTP also do not stably bind to OTP86^DYW^. When ATP- or GTP-treated OTP86^DYW^ is subjected to size-exclusion chromatography, the higher *T*_m_ of the untreated protein (inactive state) is restored in the eluate fractions; however, addition of ATP or GTP to the eluted samples leads again to a decrease of the *T*_m_, indicating a reversible structural change and a stable ground state in the absence of activators (Fig. 6a). In the size-exclusion chromatograms of isolated OTP86^DYW^ and OTP86^DYW^ pre-treated with 2 mM ATP, the A_260/280_ ratios of the respective eluted OTP86^DYW^ peaks are identical (0.54), which further supports the dissociation of the activators from the DYW domain (Supplementary Fig. 2c,d). Contrasting ATP as efficient activator, the addition of 2 mM AMP has a very mild effect. The three phosphates of ATP seem particularly important for activation as the non-hydrolysable analogue AMPPCP had only a mild effect on activation (comparable to AMP); that is, lowering the *T*_m_ to 68 °C. In summary, the activation of OTP86 seems to be either triggered by THU or triphosphate nucleotides.

To gain more insight into whether the H892C mutation effect (activity loss) is of a catalytic or structural nature, we assessed the *T*_m_ of H892C in the presence of the activators. The H892C mutant closely resembles the wild-type protein regarding activation, albeit less pronounced. The detrimental effect of H892C on activity in the in vivo assays therefore relies on the stronger zinc ligand properties of cysteine rather than an impaired structural rearrangement of the catalytic site due to activation (see Fig. 5a).

The L917A mutation showed a prominent decrease in *T*_m_ when THU was added; however, a milder effect with ATP comparable to wild type was observed. This may indicate that the activation via THU and ATP relies on different mechanisms. Finally, the R918A mutant showed a weaker decrease in *T*_m_ in the presence of activator compared with the wild-type. This may be a result of the impaired dimerization capability or an indirect destabilization of the active site via α-helix 3 and thus reduced activation (see Supplementary Fig. 8).

To consolidate and cross-validate our structural data, in vivo activities and DSF, and to gain more control about the reaction conditions, we conducted in vitro RNA editing assays with purified PPR56 and PPR56^PPRE1E2^–OTP86^DYW^ (Fig. 6b). Contrasting an earlier report, and consistent with the proposed DYW domain activation mechanism, the cytidine deaminase inhibitor THU increases deaminase activity markedly for both proteins in a concentration-dependent manner^22^. Within this study we were not able to structurally explain this effect due to the absence of THU in the electron density. In agreement with past in vitro editing assays, ATP activates PPR56 and PPR56^PPRE1E2^–OTP86^DYW^ in a concentration-dependent fashion^22^. We observe that higher ATP concentrations inhibit deaminase activity and thus confirm a highly sensitive regulation of DYW domains by ATP which may be of an allosteric type. Other trinucleotides such as GTP also activate both PPR proteins in a concentration-dependent manner, albeit with a higher sensitivity, confirming the DSF measurements. All assays cross-validate our structural data of the OTP86 DYW domain in its inactive and active states along with a complex regulation mechanism, which suggests an intricate activation of the plant organellar RNA editosome in vivo.

## Discussion

Our results draw a uniform picture of an unexpected autoinhibition mechanism elicited by DYW domains, which is released in the context of a plant RNA editosome at the site of editing. The data presented here is consistent with past in vivo mutagenesis studies and underlines the cytidine deaminase function of DYW domains in RNA editing^8,11,20,23,35,51^. Typically, cytidine deaminases are highly active enzymes^29^. With regulated DYW domains, which only exert catalysis specifically and in the context of the RNA editosome, unspecific side reactions that result in an overarching distortion of the organellar transcriptome—and lastly proteome—would be avoided. Likewise, a strict autoinhibition of the deaminase activity protects the cytosol as all RNA editosome proteins originate from nuclear transcripts and are imported into organelles^9^.

The higher target specificity of DYW type RNA editing factors in plant organelles compared with animal RNA editing deaminase enzymes suggests that the specific binding of RNA by the PPR tract can be a trigger of the DYW activation (Supplementary Note 4). It is also possible that other co-factors in the plant RNA editosome, for example MORF proteins, support moving the gating domain either directly or through changing the conformation of PPR, E1 and E2 domains.

**Fig. 7:**
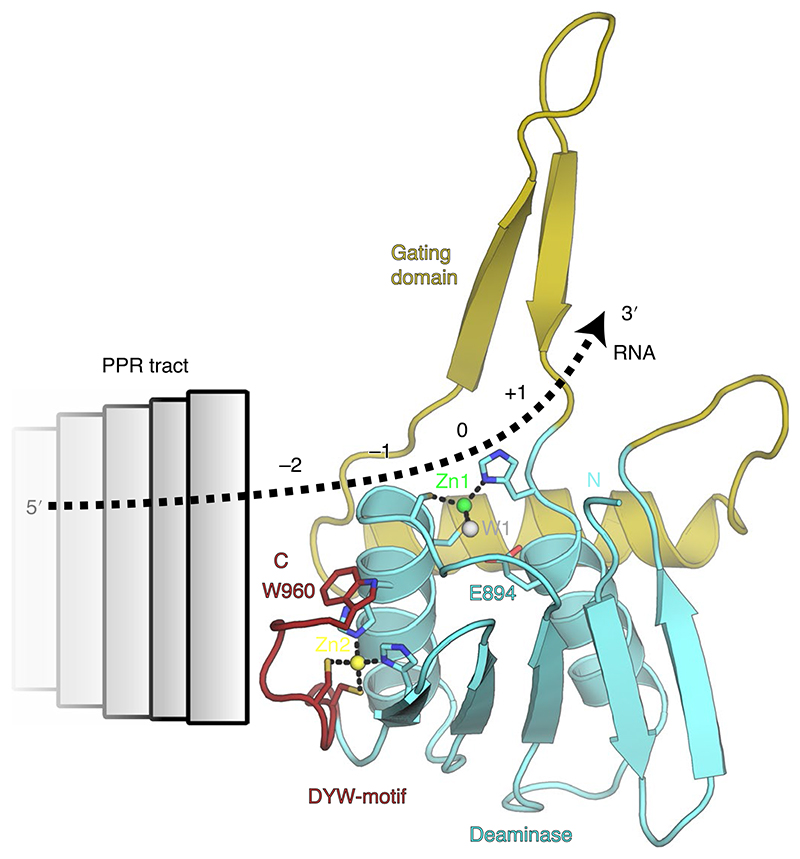
Model for the anticipated RNA path across OTP86^DYW^ in an RNA editosome context. Activation of OTP86^DYW^ is triggered by a conformational change of its gating domain, which may be elicited by an activator such as ATP, PPR–RNA complex formation, via the E1/E2 domains or different factors such as MORF proteins. The direction and path of the RNA can be extrapolated from comparisons to known cytidine deaminase co-structures such as human APOBEC3A (see Fig. 4h,i; PDB-ID: 5KEG^75^), our comprehensive mutations of the domain surface (see Fig. 5) and the charged surface of OTP86^DYW^ (see Fig. 1e). Colours and labels are as in Fig. 3a.

When we compare OTP86^DYW^ to other ligand-bound cytidine deaminases we can extrapolate that the −1 and −2 nucleotide positions relative to the editing site fall into the region of the DYW motif, indicating a head-to-tail arrangement of PPR tract and DYW domain with respect to the direction of the protein sequence of the respective proteins^32,44^ (Fig. 7). Our observations are in line with a past study in which the 0 to −3 nucleotides bind to the DYW domain, whereas the E1 and E2 motifs do not contribute to binding the target RNA^52^. In this scenario, the DYW motif bridges the PPR tract and the deaminase/gating domain, which may be the reason for its important structural role within the plant organellar RNA editosome. Furthermore, our structural data suggest a potential multimerization of DYW domains (Supplementary Note 5).

Reverse U-to-C RNA editing is observed only in hornwort, most lycophytes and ferns and might be elicited by PPR DYW:KP proteins^53–58^. Our work has several implications that this process may not depend on a strong autoinhibition (Supplementary Note 6). We searched for gating domain-like sequences in proteins of all kingdoms using a phmmer search (HmmerWeb version 2.41.1)^59^. Only PPR proteins that included a conserved gating domain sequence were detected. Finally, a comparison of members of the deaminase superfamily identified the gating domain as exclusive insertion in DYW-type PPR proteins^29^.

On the basis of our observations, we propose a regulation mechanism of RNA editing by ATP or other triphosphate nucleotides via the DYW cytidine deaminase activity. RNA editing is directly coupled to the organellar nucleotide metabolism downstream as ATP production is dependent on RNA editing. Conversely, nucleotide levels seem to regulate RNA editing, thus creating a feedback loop. In this scenario, organellar ATP synthesis and RNA editing are mutually regulated to achieve homeostasis. In the light of our artificial in vitro system with isolated proteins, we anticipate a high sensitivity of this feedback loop in vivo possibly owing to the generally low abundance of editing factors observed in mitochondria^60^.

We have further identified a very unusual regulation mechanism involving zinc coordination. In this protein regulation principle, a major domain movement alters the coordination around a zinc atom. In the inactive state, the zinc is inhibited by its coordination setting, which restricts the access of a water molecule as fourth zinc ligand required for catalysis. Upon activation, the DYW gating domain changes its conformation, which triggers the repositioning of a histidine involved in zinc coordination. The altered zinc coordination permits a water molecule to be recruited as a fourth ligand between zinc and the catalytic residue E894 to attack the base for deamination. This regulation principle may also apply to other metalloenzymes beyond DYW deaminases and we are not aware of any similar mechanism described in the current literature.

Our observations explain three decades of previously failed attempts to establish an in vitro RNA editing assay and impaired nucleotide binding of DYW domains^6,11,51^. We anticipate our results to be a valuable basis for follow-up experiments for example, a ligand-bound DYW domain structure or cryo electron microsocropy studies of a complete editosome. Based on our structure, further in vitro activity assays with structure-guided DYW domain mutants become conceivable where ligand binding, substrate binding or dimerization dependent activation is enhanced or reduced upon mutagenesis.

## Methods

### Cloning, expression and protein purification

When we set out to determine the structure of a DYW domain, we first screened 18 different *A. thaliana* (CRR22, CRR28, OTP81, OTP82, OTP84, OTP85, OTP86, OTP90, LPA66, YS1, RARE1, MEF1, MEF8, MEF10, MEF11, MEF14, MEF22 and MEF29) and three different *Physcomitrella patens* (*Pp*PPR_65, *Pp*PPR_71 and *Pp*PPR_79) DYW proteins with four different N-terminal starting points (PPR-E1E2-DYW, E1E2-DYW, DYW (according to Cheng et al.^31^) and DYW (according to Lurin et al.^61^)) and various DYW-containing constructs of nine additional DYW proteins, totalling 113 tested expression constructs. Of these, only one MEF22 and one OTP86 construct yielded small amounts of soluble protein. Only OTP86 (amino acid residues 826–960) crystallized.

A DNA fragment encoding the *A. thaliana* OTP86 DYW domain (amino acid residues 826-960) was cloned into pET28a to yield a protein (OTP86^DYW^) with a tobacco-etch-virus-cleavable (TEV-cleavable) N-terminal Strep-tag. After TEV cleavage, the protein retains the N-terminal tripeptide GAM from the tag. For protein production, *E. coli* Rosetta2 (DE3) cells were transformed with the respective plasmid, grown in terrific broth to an OD_600_ of 0.6 at 37 °C, cooled to 20 °C, induced with 0.5 mM isopropyl-β-d-thiogalactoside (IPTG) and cultivated at 16 °C overnight. Cells were harvested by centrifugation and stored at −80 °C. Cell pellets from expression cultures were resuspended in lysis buffer (20 mM Tris-HCl, pH 7.5, 200 mM NaCl), supplemented with 0,01% (w/v) CHAPS in the presence of a protease inhibitor cocktail (Roche)). Cells were lysed using a Sonoplus sonifier (Bandelin) and cell debris were removed by centrifugation. For purification of OTP86^DYW^, the soluble fraction was passed over a StrepTactin gravity flow column, pre-equilibrated with lysis buffer. The beads were washed with lysis buffer and fusion proteins were eluted with lysis buffer supplemented with 10 mM desthiobiotin. The eluate was treated with a 1:40 protein mass ratio of TEV protease (in lysis buffer) overnight to remove the N-terminal Strep-tag. Cleaved proteins were further purified via Superdex 75 gel filtration chromatography (GE Healthcare, Unicorn Software 5.20) in 20 mM Tris, pH 7.5 and 150 mM NaCl. Peak fractions of the monomers were pooled, passed over an equilibrated StrepTactin gravity flow column, concentrated to 8–15 mg ml^–1^, flash frozen in liquid nitrogen and stored at −80 °C. Any alteration to the expression construct described above (for example, variations of the N-terminus length) abolished protein solubility.

### Crystallographic analyses

OTP86^DYW^, supplemented with 2 mM UMP, crystallized by sitting drop vapour diffusion (100 nl protein plus 100 nl reservoir and 30 nl 0.1 M 50% v/v Jeffamine M-600 pH 7.0 as an additive) at 4 °C with a reservoir containing 0.1 M glycine, pH 10.5, 1.2 M NaH_2_PO_4_, 0.8 M K_2_HPO_4_ and 0.2 M Li_2_SO_4_ (space group *C*2). Crystals were cryoprotected with reservoir solution supplemented with 15% (v/v) ethylene glycol. Diffraction data to 2.5 Å resolution were collected at 100 K at beamline 14.1 of the BESSY II storage ring^62^. All diffraction data were processed with XDS^63^. Activated OTP86^DYW*★*^—supplemented with 2 mM CMP and 2 mM THU—crystallized by sitting drop vapour diffusion (1 μl protein plus 1 μl reservoir) in 100 mM sodium acetate (pH 4.6) and 2 M sodium formate (space group *P*2_1_2_1_2), with a pronounced degree of translational non-crystallographic symmetry. Crystals were cryoprotected with reservoir solution supplemented with 2 mM CTP, 2 mM THU, and adjusted to a concentration of 3 M sodium formate as a cryoprotectant. Diffraction data to 1.65 Å resolution were collected at 100 K at beamline 14.1 of the BESSY II storage ring^62^. All diffraction data were processed with XDS^63^.

The structure of OTP86^DYW^ was solved by single-wavelength anomalous dispersion with four zinc sites in space group *C*2 and two molecules per asymmetric unit employing PHENIX.AUTOSOL^64^. The initial density modified map was iteratively improved by manual model building with Coot^65^ and refined with PHENIX.REFINE (including experimental phases in the initial stages); automated model building was performed with PHENIX.AUTOBUILD^64–66^. The structure of OTP86^DYW*★*^ was solved by molecular replacement with PHASER^67^ employing the structural coordinates of a truncated OTP86^DYW^, encompassing the deaminase domain and DYW motif. Despite the translational non-crystallographic symmetry, structure solution and refinement were successful, albeit with slightly increased R-factors (see Supplementary Table 1). The remaining model parts were built manually with COOT^65^ and with PHENIX.AUTOBUILD^64–66^ in an iterative fashion to improve the model until completion. Structure figures were rendered with open source Pymol v.1.8, structural movies were made with Pymol 2.2.3 (Schrödinger) under an academic license. Electrostatic surface potential was obtained by APBS employing a Pymol addon^68–70^. C-alpha r.m.s.d. values for structural comparison were calculated with CCP4i^71^.

### DSF

The DSF experiments were performed in a 96-well plate in a plate reader combined with a thermocycler (Stratagene Mx3005P). Purified OTP86^DYW^ or mutants were diluted to 0.2 mg ml^–1^ in buffer A (20 mM Tris, pH 7.5; 150 mM NaCl) supplemented with 10× SYPRO orange (1:500 dilution of the stock) in a total volume of 10 μl and pipetted into a 96-well plate. Either 10 μl of buffer A or 10 μl of buffer A supplemented with the respective ligand were added to the SYPRO orange/protein mixture. The temperature was increased from 25 °C to 95 °C and the fluorescence emission was monitored in steps of 1 °C per min with hold steps of 30 s between reads. The fluorescence intensity was then plotted as a function of temperature. The sigmoidal curve from each condition was normalized and corrected for the background signal of the fluorophore in the buffer. The inflection points of the curves, representing the thermal melting temperature of the protein in the respective conditions, were compared. Each experiment was done in triplicate, averaged and a standard deviation of the respective melting temperatures was calculated.

### Size-exclusion chromatography

OTP86^DYW^ was analysed by analytical size-exclusion chromatography on a Superdex 75 PC3.2 column (GE Healthcare, Unicorn Software 5.20) in size-exclusion buffer (20 mM Tris-HCl, pH 7.5, 150 mM NaCl) at a flow rate of 50–70 μl min^–1^. Eluted fractions were analysed by sodium dodecyl sulfate–polyacrylamide gel electrophoresis (SDS–PAGE) or subjected to DSF. Calibration chromatograms for the column were obtained from GE healthcare online support.

### Cloning of MBP-PPR56^PPRE1E2^-OTP86^DYW^ and its OTP86^DYW^ mutants

Plasmids containing wild-type Physcomitrium PPR56 (pETG41K::PPR56) were previously described in ref. ^23^. For pETG41K::PPR56^PPRE1E2^–OTP86^DYW^, DNA fragments for PPR domain, E1 and E2 domains of PPR56 (amino acid residues 213–556) and DYW domain of OTP86 (825–960) were separately amplified by polymerase chain reaction (PCR) and cloned into the BsrGI digested pETG41K::PPR56 with NEBuilder (New England Biolabs). For cloning mutants, the C-terminal part OTP86 DYW was amplified by PCR with a mutation introduced primer set, and the remainder of PPR56^PPRE1E2^–OTP86^DYW^ was separately amplified to create a 15 bp overlap to the mutated PCR fragments. The two PCR fragments were simultaneously cloned into the BsrGI digested pETG41K::PPR56 by NEBuilder (New England Biolabs). The mutated OTP86^DYW^ in pETG41K was amplified by PCR and cloned into pET28a for the OTP86^DYW^ mutant protein expression constructs.

### In *E. coli* RNA editing assay

Expression of recombinant PPR proteins was performed as previously described in ref. ^23^. Plasmids were transformed into *E. coli* BL21(DE3) (TAKARA), and 5 ml *E. coli* starter cultures (lysogeny broth medium with 50 μM kanamycin) were grown overnight; 40 μl of the pre-culture was transferred to 4 ml of the same media supplemented with 0.4 mM ZnSO_4_ in a 15 ml cell culture tube (IWAKI, http://www.atgc.co.jp). Cultures were grown at 37 °C until an OD_600_ of 0.4–0.7 was reached. Cultures were cooled on ice for 5 min before adding 0.4 mM IPTG for induction of construct expression. Cells were incubated at 180 rpm at 16 °C for 20 h. After stopping induction, 2 ml of the sample was transferred to a sample tube for SDS–PAGE, with another 1 ml to a tube for RNA editing analysis. The respective samples were harvested and the pellets were frozen in liquid nitrogen and stored at −80 °C until further use. For the RNA editing assay, the total RNA was extracted from the *E. coli* cells after adding 100 μl of the lysozyme buffer (10 mM Tris-HCl (pH 8.0), 0.1 mM EDTA, 10 mg ml^–1^ lysozyme) using a Maxwell RSC Plant RNA Kit system (Promega, www.promega.com). Isolated RNA was used for PCR with reverse transcription (RT–PCR). Data were analysed with Microsoft Excel and plotted with Python/Matplotlib.

### Validation of the amount of mutated recombinant proteins in *E.coli*

*Escherichia coli* cells from 2 ml culture were resuspended in 1 ml of chilled lysis buffer (50 mM Tris-HCl, pH 7.5, 300 mM NaCl, 10% glycerol, 5 mM imidazole, 0.07% mercaptoethanol, 0.1% Triton-X-100, 1× complete EDTA-free (Roche) and 1 mM PMSF) and the soluble fraction was isolated after sonication and centrifugation; 7.5 μl of the soluble protein lysate was loaded on SDS-PAGE gels for silver staining (Source Data for Supplementary Fig. 7). For the western blot analysis, 150 μl of the soluble protein lysate was precipitated with 400 μl acetone. After centrifugation at 4 °C for 30 min at 15,000 rpm, the pellet was extracted with 15 μl of 1× loading buffer (50 mM Tris-HCl (pH6.8), 2% SDS, 0.2% bromophenol blue, 100 mM DTT, 10% glycerol) and loaded onto an SDS-PAGE gel. Expression of recombinant proteins was assayed by western blot analysis with an anti His-Tag antibody (PGI proteintech Group; AB_11232599) at 1:20,000 dilution followed by incubation with Anti-Mouse IgG, HRP-Linked Whole Ab (GE Healthcare; AB_772209) at 1:50,000 dillution. Signals were detected with ECL Prime Western Blotting Detection Reagent (GE Healthcare) and visualized with an ImageQuant LAS4000 (GE Healthcare). The signal intensities of the western blot analysis were analysed using ImageQuant TL v.8.1 (GE healthcare).

### Expression of PPR proteins in *E.coli* for in vitro assays

Lysogeny broth medium (50 ml) with 50 μM kanamycin was inoculated with 500 μl of overnight cultures and incubated at 37 °C for 2 h to an OD_600_ of around 0.4–0.6. The cultures were cooled on ice for at least 5 min and ZnSO_4_ was added to a final concentration of 0.4 mM and IPTG to 0.4 mM to induce expression. Cells were incubated at 16 °C and 180 rpm for 20 h. The cells were centrifuged at 4 °C, 5,000 rpm for 10 min and cell pellets were suspend in 5 ml lysis buffer (50 mM Tris-HCl (pH 7.5), 200 mM NaCl, 0.07% mercaptoethanol and 1 mM phenylmethylsulfonyl fluoride). The *E. coli* cells were sonicated with six sets of 10 × 2 s pulses with 1 min breaks while on ice. After centrifugation at 4 °C for 10 min at 15,000 rpm, the supernatant from the 5 ml samples was mixed with 30 μl of amylose resin (New England Biolabs) equilibrated in lysis buffer and mixed with the rotary machine for 1 h at 4 °C. The amylose resin was washed three times using 1 ml of lysis buffer. Proteins were eluted with 30 μl of elution buffer (lysis buffer with 10 mM maltose).

### Preparation of RNA editing substrates

Polymerase-chain-reaction fragments were amplified using the pETG41K::PPR56 as a template, and primers nad4FEcoRV:GGCCTCTTGCGGGATATCTCAAACA TCAATTTTTATATAGGTATAGACGGTATCT and nad4RBamH: CCGGCGTAGAGGATCCAAAATGAAGAGATACCGTCTATACCTATA. This fragment was cloned into pACYC184 digested with EcoRV and BamHI by NEBuilder. Furthermore, using this clone (pACYC184-Ppnad4) as a template, a PCR amplicon was synthesized with primers T7KS_pACY184EF: GTAATACGACTCACTATAGGGCTCGAGGTCGACGGTATCAATCTAACA ATGCGCTCATC and SKR-pACYC184_EB_R: CGCTCTAGAACTAGTGGATCCAGCGACGGAATCTTACTTA produced amplicons with a 5′ T7 promoter sequence. The amplicon was purified with a PCR purification kit (QIAGEN). RNA was synthesized with T7 polymerase (TAKARA) using the PCR amplicon as a template. RNA was diluted to 100 fmol μl^–1^ and used for the reaction with purified recombinant proteins.

### In vitro RNA editing activity assay

Standard in vitro RNA editing reaction mixtures contained 100 mM Tris-HCl (pH 7.5), 10 mM maltose, 0.017%mercaptoethanol, 10U of RNaseOUT (Invitrogen), 1× proteinase inhibitor mixture complete EDTA-free (Roche), 100 fmol of mRNA substrate, and 2.5 μg of purified recombinant PPR56 proteins (PPR56^PPRE1E2^–OTP86^DYW^) or its mutated variants. The reaction mixtures were incubated at 16 °C for 2.5 h and purified RNAs were used for RT–PCR reactions.

### Detection of C-to-U RNA editing

Complementary DNA was synthesized with a random hexamer with ReverTra Ace qPCR RT Master Mix with gDNA Remover (TOYOBO) for both in *E. coli* and in vitro editing assays. A reverse primer upstream of the T7 terminator sequence and a forward primer binding the PPR56 coding region for in *E. coli* assay and KS and SK primers for the in vitro assay were used for RT–PCR amplification with GoTaq Master Mixes (Promega). After 5 min initial denaturation at 94 °C followed by 35 cycles each with 30 s denaturation at 94 °C, 30 s annealing at 55 °C, 1 min synthesis at 72 °C. For purification of PCR products, 2U ExoI (TAKARA) and 0.5U Shrimp Alkaline Phosphatase (TAKARA) were added and incubated at 37 °C for 1 h followed by 15 min at 80 °C and sequenced directly (Macrogen, www.macrogen-japan.co.jp or GENEWIZ, https://www.genewiz.com). Sequencing chromatograms were analysed with DNADynamo v.1.608 (www.bluetractorsoftware.co.uk). RNA editing was quantified as the ratio of the resulting thymidine peak to the sum of the thymidine and cytidine peak heights at the respective editing site. Editing values are given as the mean of at least three replicates with standard deviations. Data were analysed with Microsoft Excel and plotted with Python/Matplotlib.

### Reporting summary

Further information on research design is available in the Nature Research Reporting Summary linked to this article.

## Supplementary Material

Supplementary Information

## Data Availability

Structure coordinates and diffraction data were deposited with the Protein Data Bank (http://www.pdb.org) under accession codes 7O4E (OTP86^DYW^) and 7O4F (OTP86^DYW*★*^). Source data are provided with this paper. The data that support the findings of this study are available from the corresponding authors on reasonable request.
