# Supplementary material for: DYW domain structures imply an unusual regulation principle in plant organellar RNA editing catalysis
Source: Nat Catal. Author manuscript; Available in PMC 2021 Oct 27. (PMC7611903; doi:10.1038/s41929-021-00633-x)
Supplement: Supplementary Information [file EMS136450-supplement-Supplementary_Information.pdf]

nature portfolio

https://doi.org/10.1038/s41929-021-00633-x

Supplementary information

DYW domain structures imply an unusual regulation principle in plant organellar RNA editing catalysis

In the format provided by the authors and unedited

Supplementary Information for

DYW domain structures imply an unusual regulation principle in plant organellar RNA editing catalysis

Mizuki Takenaka[1,*], Sachi Takenaka[1,§], Tatjana Barthel[2,#,§], Brody Frink[1], Sascha Haag[3], Daniil Verbitskiy[3], Bastian Oldenkott[4], Mareike Schallenberg-Rüdinger[4], Christian Feiler[5], Manfred S. Weiss[5], Gottfried J. Palm[2] and Gert Weber[2,#,*]

Affiliations:

[1] Department of Botany, Graduate School of Science, Kyoto University, Oiwake-cho, Sakyo-ku, Kyoto, 606-8502

[2] University of Greifswald, Molecular Structural Biology, Felix-Hausdorff-Str. 4 17487 Greifswald, Germany

[3] Molekulare Botanik, Universität Ulm, Albert-Einstein-Allee 11, 89069 Ulm, Germany.

[4] IZMB – Institut für Zelluläre und Molekulare Botanik, Abt. Molekulare Evolution, University of Bonn, Kirschallee 1, 53115 Bonn, Germany

[5] Helmholtz-Zentrum Berlin für Materialien und Energie, Macromolecular Crystallography, Albert-Einstein-Straße 15, D-12489 Berlin, Germany

[#] Present address: Helmholtz-Zentrum Berlin für Materialien und Energie, Macromolecular Crystallography, Albert-Einstein-Straße 15, D-12489 Berlin, Germany

* Correspondence and requests for materials should be addressed to M.T. (mizuki.takenaka@pmg.bot.kyoto-u.ac.jp) or to G.W. (gert.weber@helmholtz-berlin.de)

[§] These authors contributed equally

Table of contents

Supplementary Figure 1 3
Source Data for Supplementary Figure 1b 4
Supplementary Figure 2 5
Source Data for Supplementary Figure 2a 6
Source Data for Supplementary Figure 2b 7
Supplementary Figure 3 8
Source Data for Supplementary Figure 3a 9
Supplementary Figure 4 10
Supplementary Figure 5 11
Supplementary Figure 6 12
Supplementary Figure 7 13
1.) Silver staining images for *E.coli* proteins used for the western blot analysis. 14
2.) Uncropped western blot images. 15
3.) Specificity validation for Anti-6 x His-tag and Anti-Mouse IgG Anti-Mouse IgG. 18
Supplementary Figure 8 19
Supplementary Table 1 20
Supplementary Table 2 21
Supplementary Note 1 22
Supplementary Note 2 22
Supplementary Note 3 22
Supplementary Note 4 23
Supplementary Note 5 23
Supplementary Note 6 23
Supplementary References 24

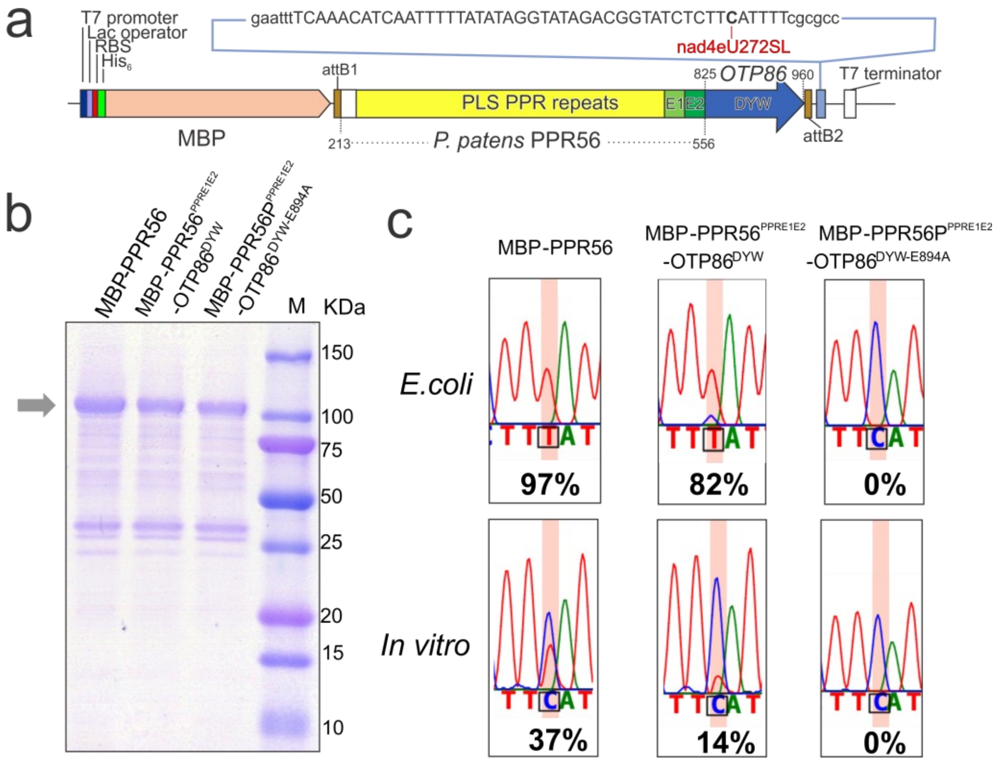

Supplementary Figure 1 OTP86^{DYW} has cytidine deaminase activity and can be purified fused to a PPR tract. **(a)** PLS PPR, E1 and E2 domains of Physcomitrium PPR56 (213-556) is fused to the OTP86 DYW domain (825-960) and inserted into the pETG_41K vector. The chimeric PPR protein is expressed as His$_6$-tagged maltose binding protein (MBP) fusion. The editing target sequence cloned downstream is co-expressed with the chimeric PPR gene. The editing site is labelled with target gene name (nad4) followed by 'eU', nucleotide position from the start codon and resulting amino acid change. Expression is driven by a T7 promoter with a lac operon and inducible by IPTG. RBS: Ribosome binding site **(b)** SDS-PAGE analysis of purified MBP fused PPR proteins used for the in vitro deamination assay. An arrow indicates the predicted size of the recombinant protein (121kDa). Purification of the recombinant proteins was repeated at least three times with similar results. M – Protein standard **(c)** Physcomitrium PPR56 is one of the two so far available RNA editing factors for orthogonal in *E. coli* editing assays. N-terminal Maltose Binding Protein (MBP) fused PPR56 (MBP-PPR56) edited the co-expressed target cytidine (nad4eU272SL). Substitution of the DYW domain of PPR56 for that of OTP86 (MBP-PPR56^{PPRE1E2}-OTP86^{DYW}) also edited the target site (Fig. 5a). Mutation at the conserved catalytic amino acid residue E894 (MBP-PPR56 ^{PPRE1E2}-OTP86$^{DYW\text{-}E894A}$) abolished the activity. Purified recombinant PPR56 as well as PPR56 ^{PPRE1E2}-OTP86^{DYW} also showed in vitro cytidine deamination activity while the PPR56^{PPRE1E2}-OTP86$^{DYW\text{-}E894A}$ mutant does not (Fig. 6b).

Source Data for Supplementary Figure 1b

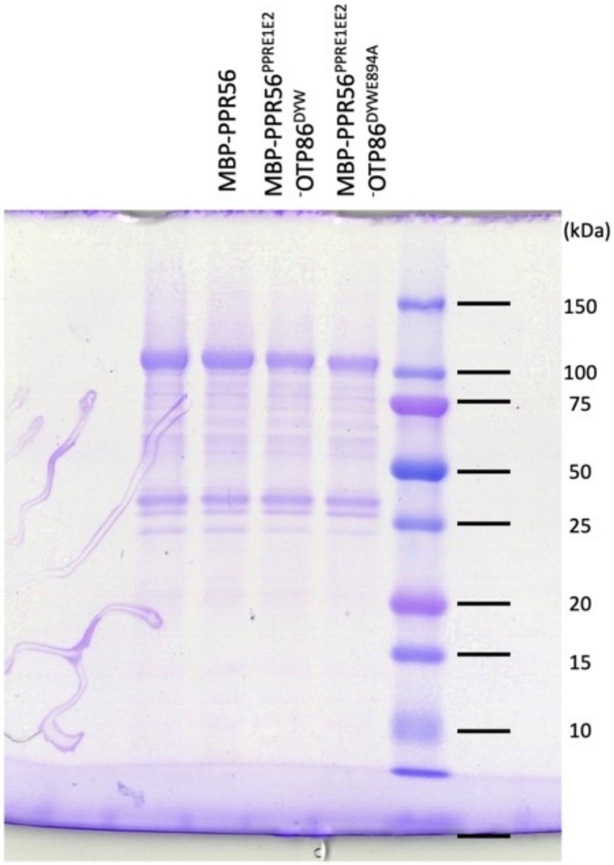

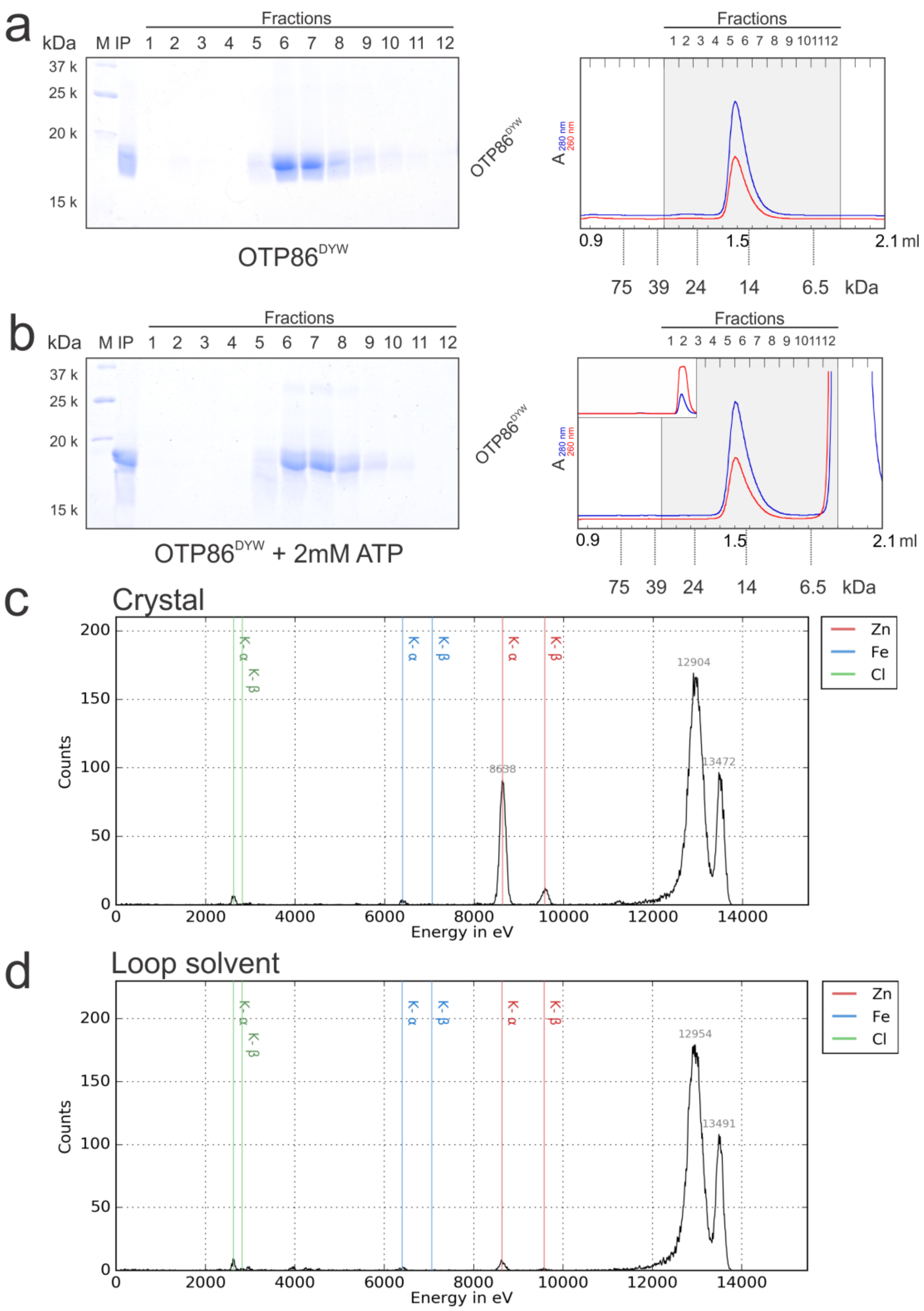

Supplementary Figure 2 Characterization of OTP86^{DYW} by size exclusion chromatography and X-ray fluorescence spectroscopy. **(a)** SDS-PAGE analysis of purified OTP86^{DYW} and **(b)** purified OTP86^{DYW} supplemented with 2 mM ATP analysed by size exclusion chromatography. Left – SDS-PAGE analysis of the eluted gel filtration fractions (indicated at the top). The first two lanes show the protein standard (M) and the gel

filtration input (IP). Molecular mass of the standard proteins in kDa are shown on the left, protein names on the right. Gel filtrations were repeated independently one time with similar results. Right – Elution profiles of the respective gel filtration runs. The absorption values were scaled to fit the OTP86^{DYW} peak while maintaining the AU_{260}/AU_{280} ratios. The upper left inset in (b) shows the absorption values scaled to the larger ATP peak. Fractions corresponding to the SDS PAGE analysis are highlighted in grey, fraction numbers are shown at the top, elution volumes at the bottom of the profile. Dashed lines across the elution profiles indicate the elution peaks of molecular mass standards (molecular masses given at the bottom). **(c)** X-ray fluorescence spectrogram of an OTP86^{DYW} crystal displayed and analysed using the program XFEPLOT (https://www.helmholtz-berlin.de/forschung/oe/np/gmx/xfeplot/index_en.html). A crystal of OTP86^{DYW} was exposed to an X-ray beam of 13.5 keV energy on the HZB-MX beamline BL14.2 and the resulting fluorescence was recorded using a fluorescence detector[1]. **(d)** X-ray fluorescence spectrogram of loop solvent corresponding to (c) displayed and analysed as in (c). The Fe-signal in (c) and (d) is likely derived from the sample holder, the sample may also contain traces of Cl.

Source Data for Supplementary Figure 2a

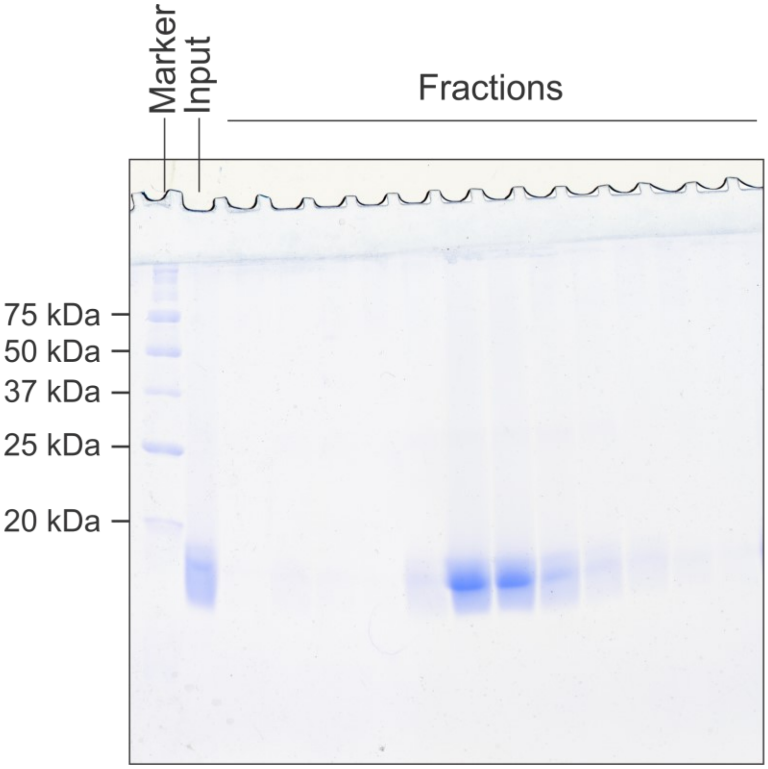

Source Data for Supplementary Figure 2b

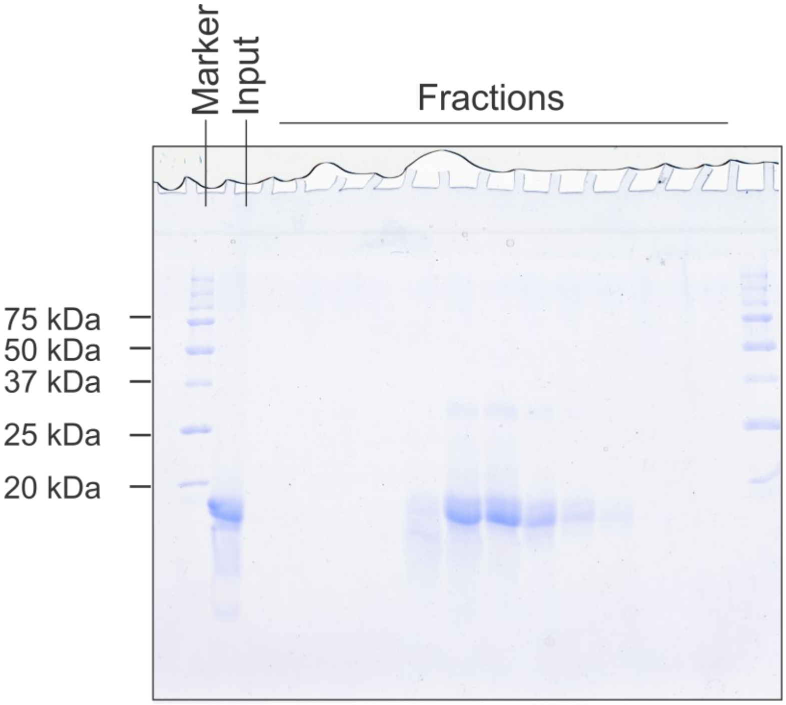

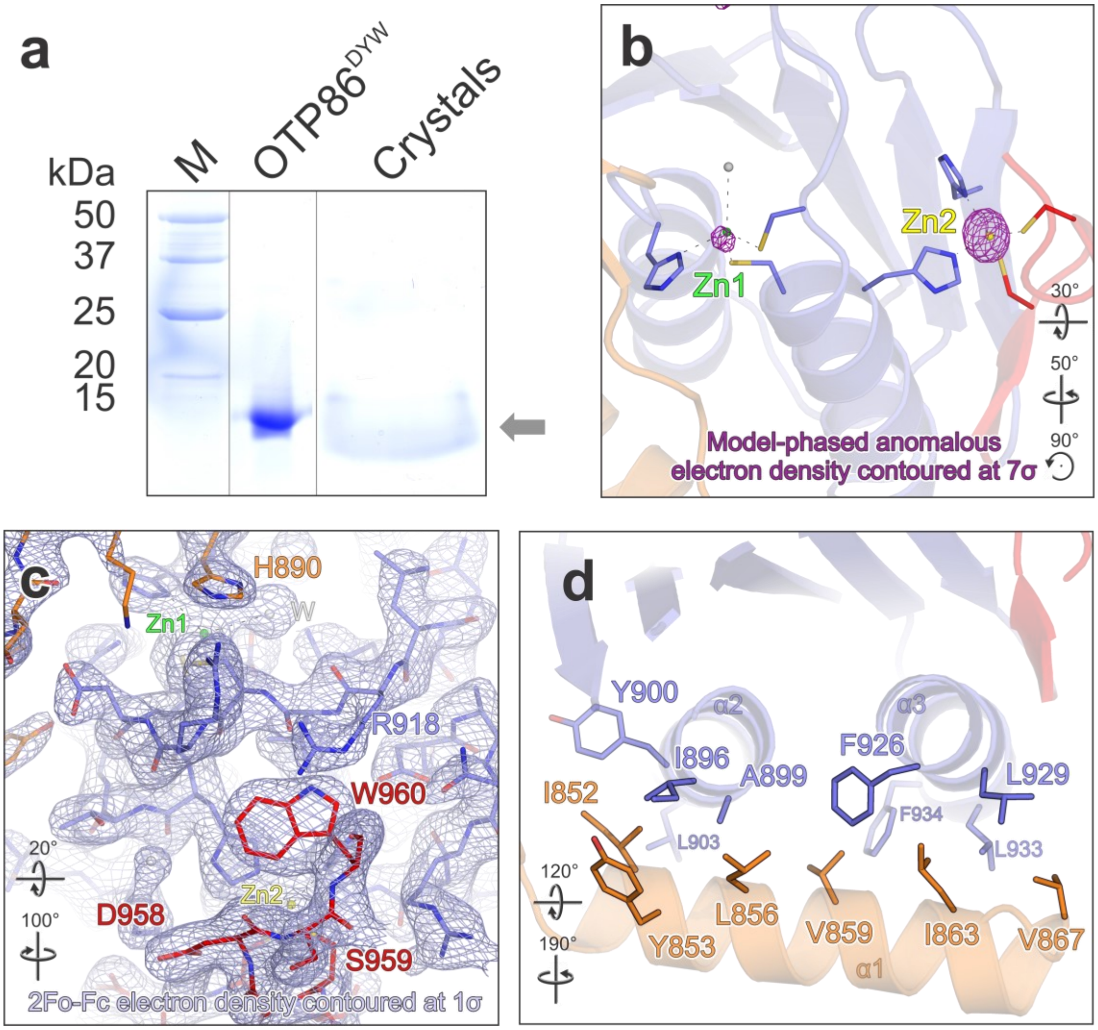

Supplementary Figure 3 SDS PAGE analysis of *A. thaliana* OTP86^{DYW} crystals model-phased anomalous and final 2F$_O$-F$_C$ electron density. **(a)** SDS PAGE analysis of OTP86^{DYW} and washed and dissolved crystals of OTP86^{DYW}. The migration behaviour of the dissolved crystals is likely altered by the residual crystallization buffer (2 M phosphate) in the sample. Lanes were re-arranged, i.e., empty lanes were cut out of the gel. Characterization of the crystals by SDS PAGE was repeated independently once with similar results. M – Protein standard **(b)** An anomalous electron density map (purple) was calculated with model-phases and contoured at 7σ. Both coordinated Zn atoms and residues or water molecules relevant for coordination are shown as sticks and spheres, respectively. Relevant residues are labelled. **(c)** The final 2F$_o$-F$_c$ electron density map of the inactive DYW domain (steel blue mesh) covering the active site and the DYW motif, contoured at 1 σ. Water molecules are shown as white spheres. **(d)** The amphipathic α-helix 3 of the gating domain spans orthogonally across both cytidine deaminase α-helices. Interface residues as sticks. Colours and labelling as in Fig. 1b. Rotation symbols indicate the views relative to Fig.1b.

Source Data for Supplementary Figure 3a

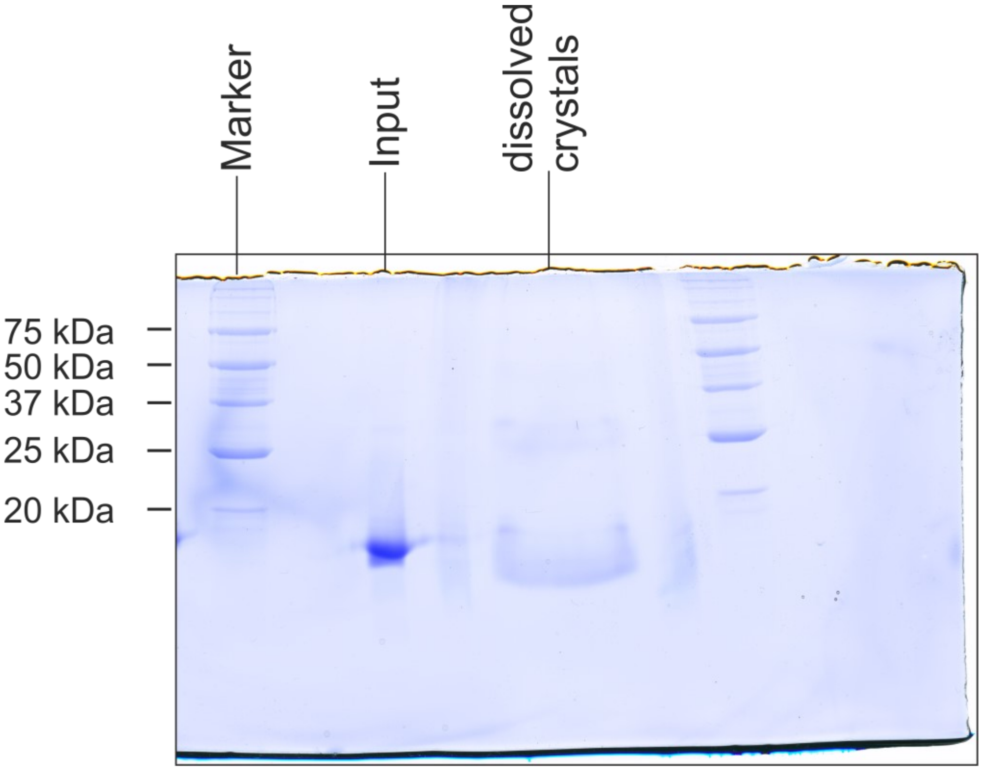

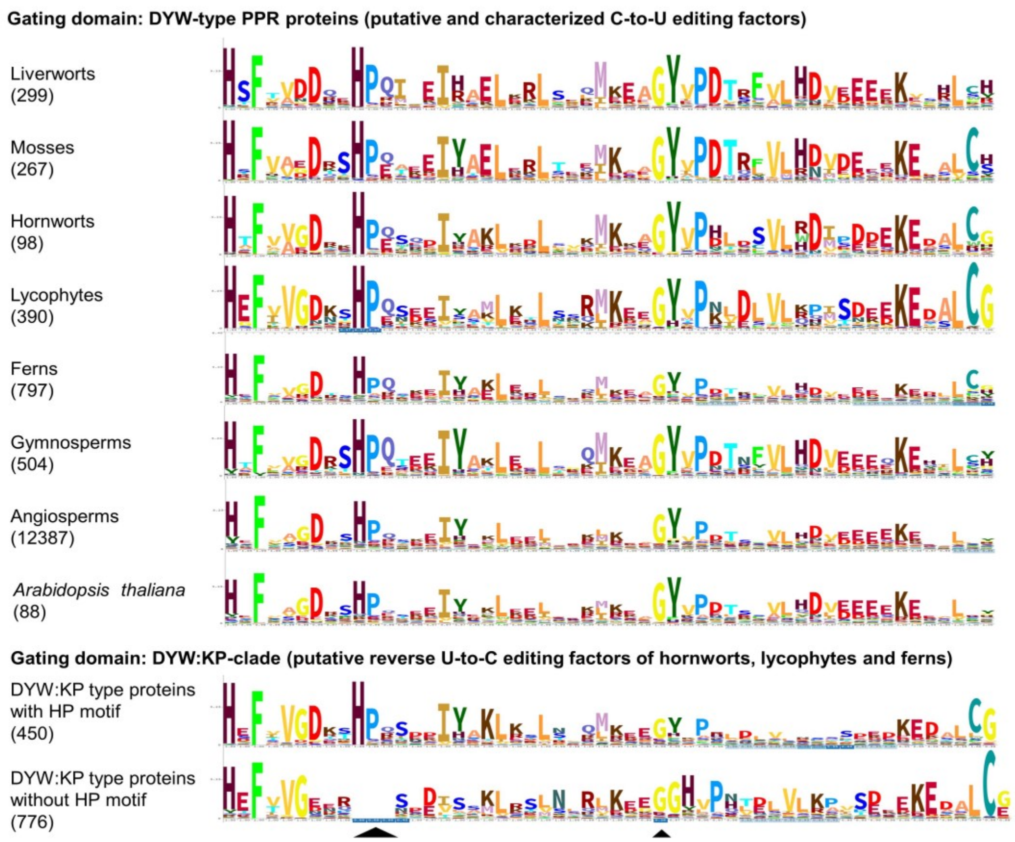

Supplementary Figure 4 The gating domain is conserved in DYW domains of PPR proteins of all major land plant clades. Organisms outside of the land plants with DYW-type PPR proteins or DYW (and gating) domains encoded in their genome are rare exceptions[2–5]. DYW domains of DYW-type PPR proteins of liverworts, mosses, hornworts, lycophytes, ferns, gymnosperms and angiosperms (OneKP dataset[2], https://ppr.plantenergy.uwa.edu.au/onekp/, see Source Data, A-J) were aligned using Mega 7[6] and the Muscle alignment algorithm[7] or MAFFT[8]. DYW domains of mosses *Physcomitrium patens* (10), *Funaria hygrometrica* (9) and angiosperm *Arabidopsis thaliana* (88) were additionally included in the analysis[3,9]. Amino acid insertions in less than 10% of the aligned gating domain sequences of each clade were excluded. Conservation logos of gating domains of each land plant clade were generated based on the alignments (see Source Data, K-T) using Skylign[10]. Stack height indicates information content, with the letter height within each stack indicating the probability of each amino acid at that position. For DYW-type PPR proteins of the DYW:KP clade, exclusively present in hornworts, lycophytes and ferns and proposed as U-to-C editing factors[2,11], separate logos were generated. DYW-type PPR proteins of all land plant clades share a gating domain with same conservation patterns. More than half of the gating domains of the DYW proteins of the DYW:KP clade show a three to four amino acid deletion including the conserved HP motif (without HP, broad black triangle). A glycine residue is additionally duplicated in ~20% of them (black triangle).

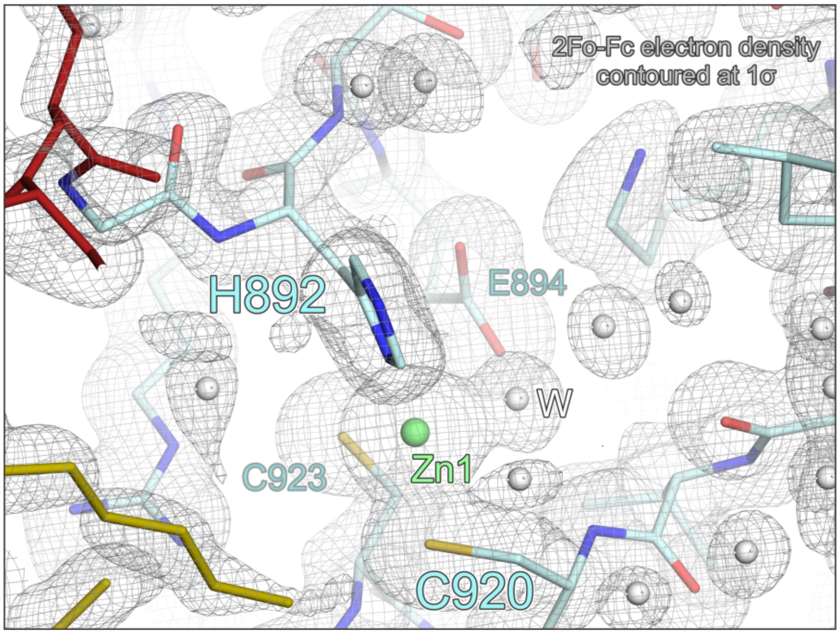

Supplementary Figure 5 The final 2Fo-Fc electron density map of the activated DYW* domain (grey mesh) covering the active site, contoured at 1 σ. Water molecules are shown as white spheres. Colours as in Fig. 3a.

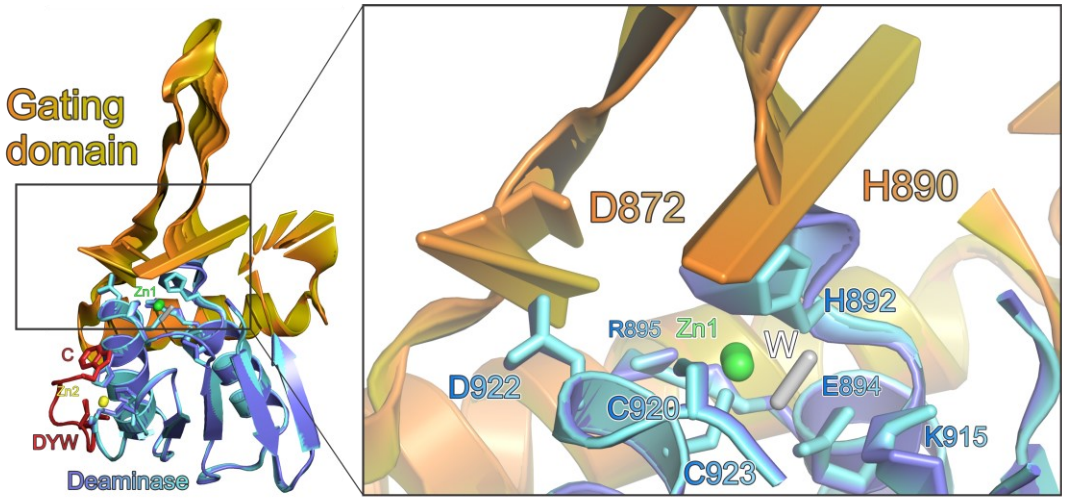

Supplementary Figure 6 Structural changes of OTP86^{DYW} upon activation. Hypothetical coordinate trajectories of backbone ribbon and selected side chains in the activation process were plotted employing a colour gradient from the inactive state (deaminase: slate, gating domain: orange, DYW domain: red) to the active state (deaminase: cyan, gating domain: sand, DYW domain: firebrick). The inset in the right panel shows a closeup of the active site. Colouring as in Fig. 3b, c.

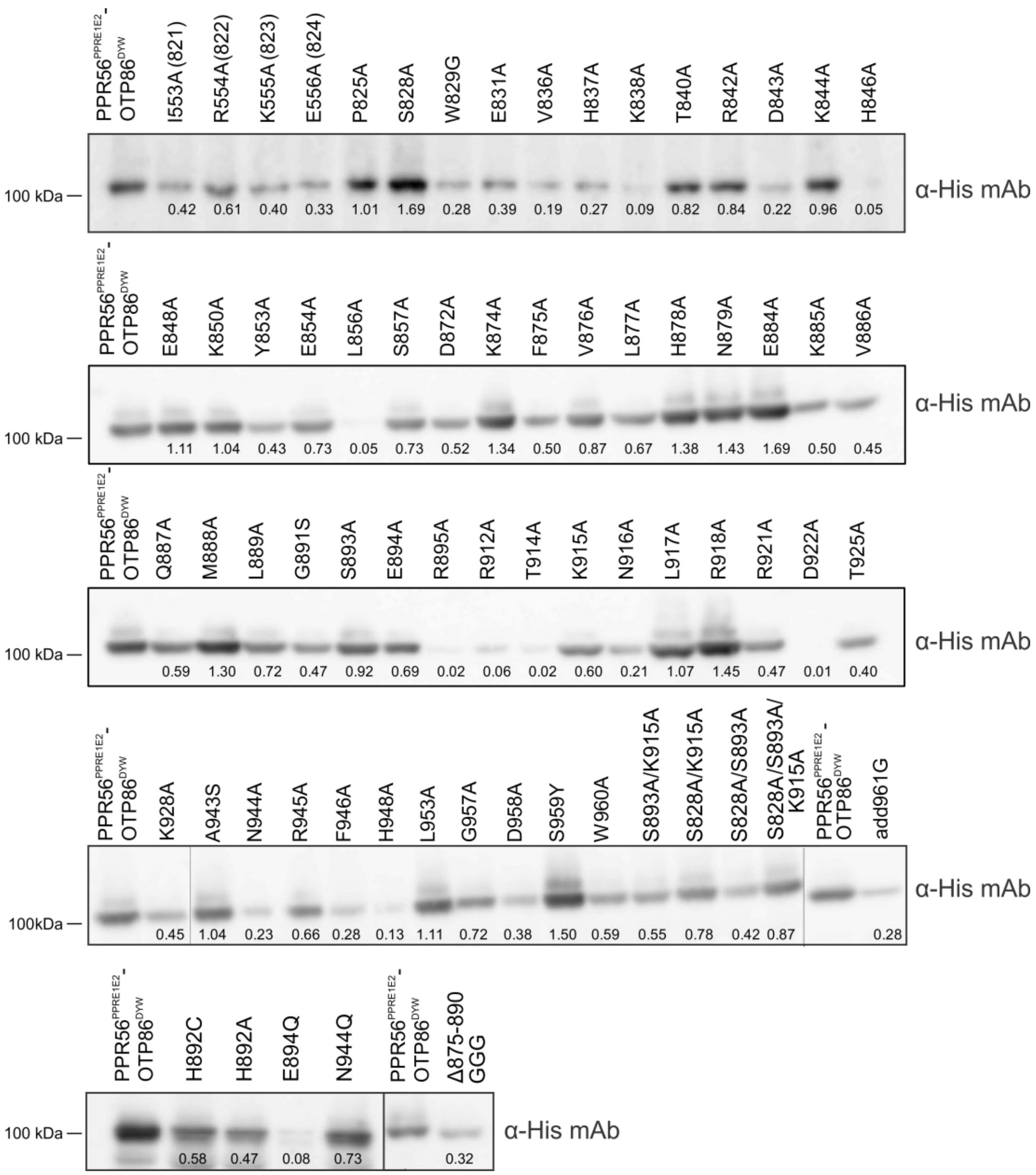

Supplementary Figure 7 Western blot analyses using an anti-hexahistidine antibody for evaluating the amounts of soluble PPR56 ^{PPRE1E2}-OTP86^{DYW} mutant proteins in the *E. coli* editing assays (Fig. 5). The numbers below the signals indicate relative signal intensity to the control PPR56^{PPRE1E2}-OTP86^{DYW}. While very low amounts of soluble proteins are sufficient for comparable RNA editing in *E. coli* (e.g. K838A, L856A, D922A), some mutants show low or no detectable editing activity in spite of accumulation of soluble proteins in *E. coli* (e.g. K555A(823), H892A, H892C, E894A, L917A, R918A, W960A). Western blot analysis was done once with a control of protein amounts by silver staining.

Source Data for Supplementary Figure 7.

1.) Silver staining images for *E.coli* proteins used for the western blot analysis.

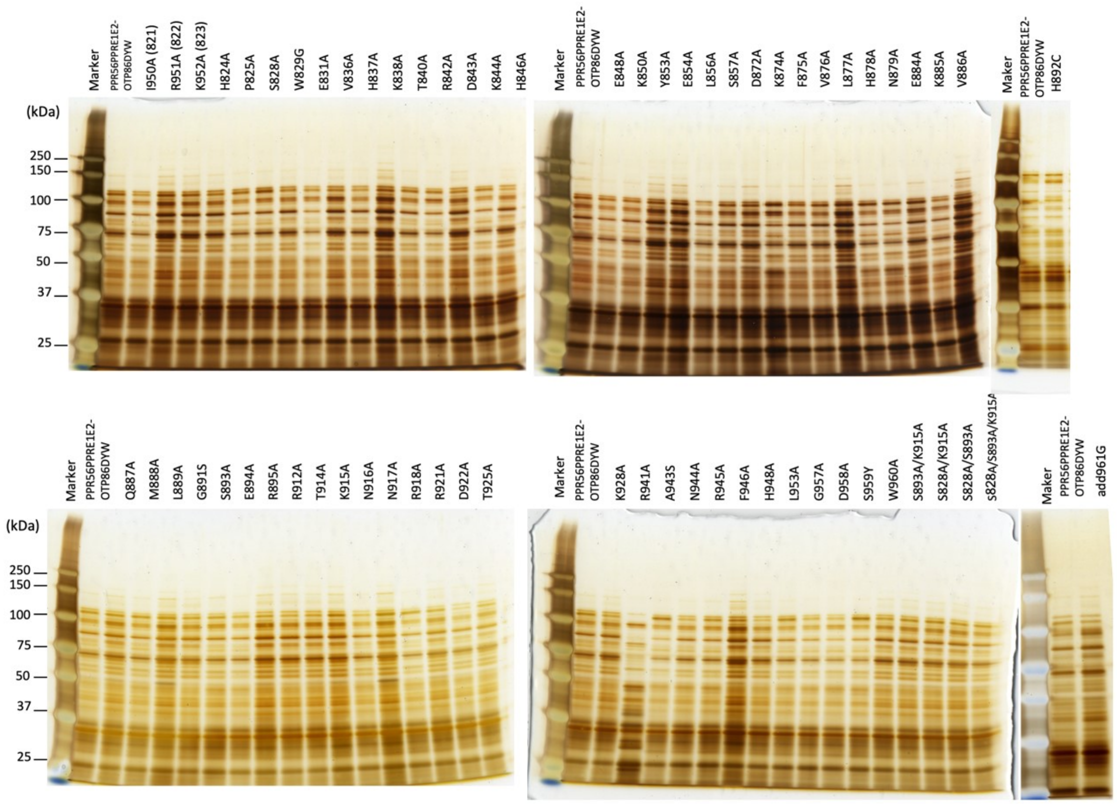

2.) Uncropped western blot images.

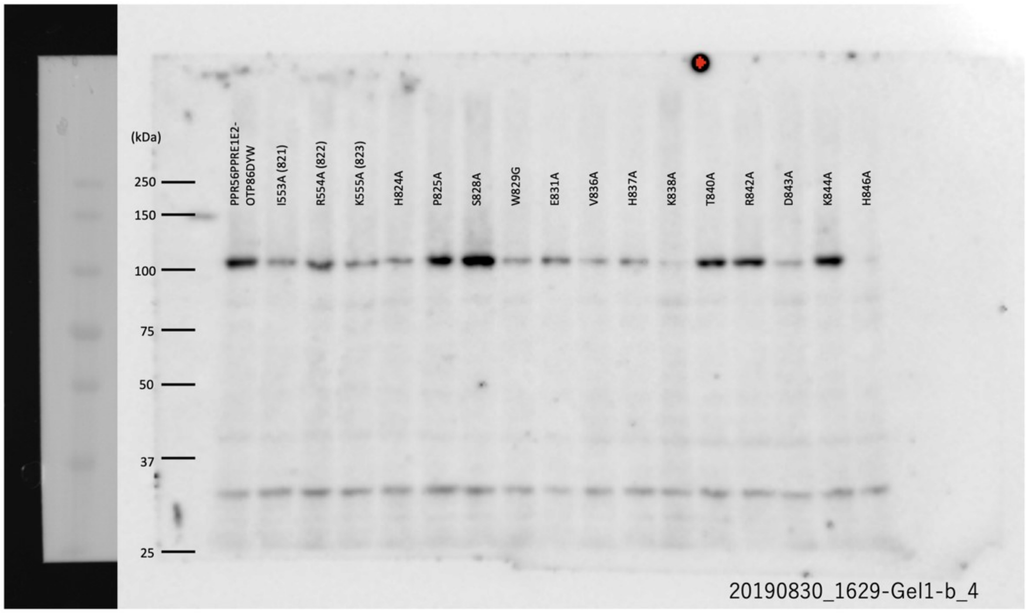

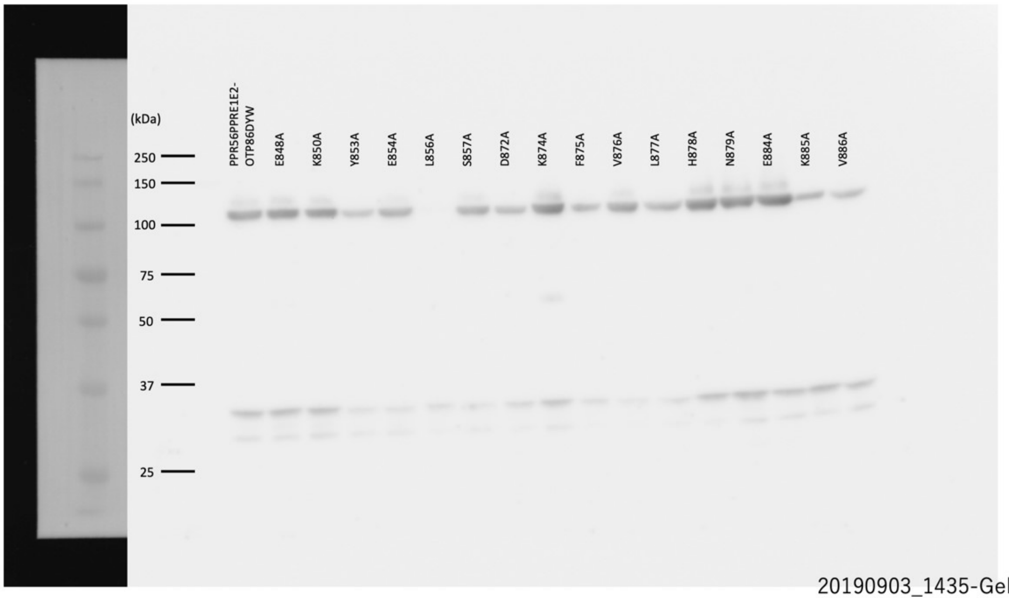

20190903_1435-Gel2-a_2d

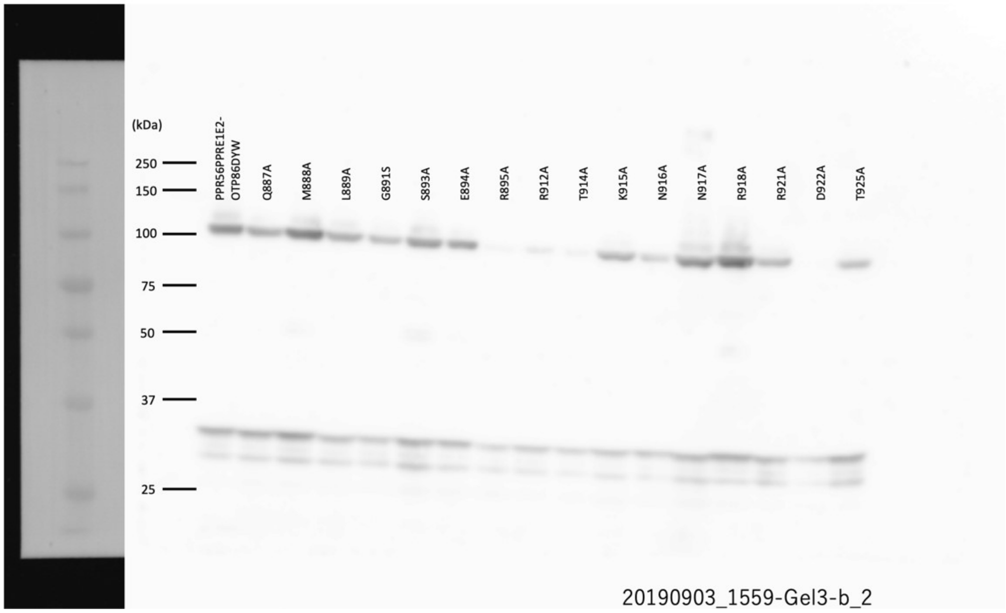
(kDa)
250
150
100
75
50
37
25
PPR56PPRE1E2-OTP86DYW
Q887A
M888A
L889A
G891S
S893A
E894A
R895A
R912A
T914A
K915A
N916A
N917A
R918A
R921A
D922A
T925A
20190903_1559-Gel3-b_2

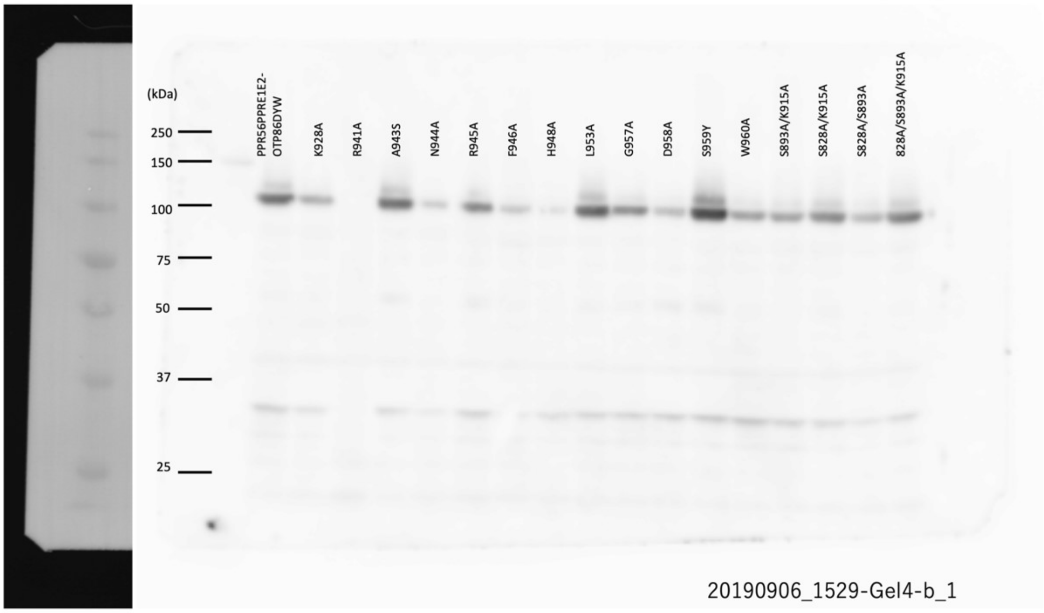
(kDa)
250
150
100
75
50
37
25
PPR56PPRE1E2-OTP86DYW
K928A
R941A
A943S
N944A
R945A
F946A
H948A
L953A
G957A
D958A
S959Y
W960A
S893A/K915A
S828A/K915A
S828A/S893A
828A/S893A/K915A
20190906_1529-Gel4-b_1

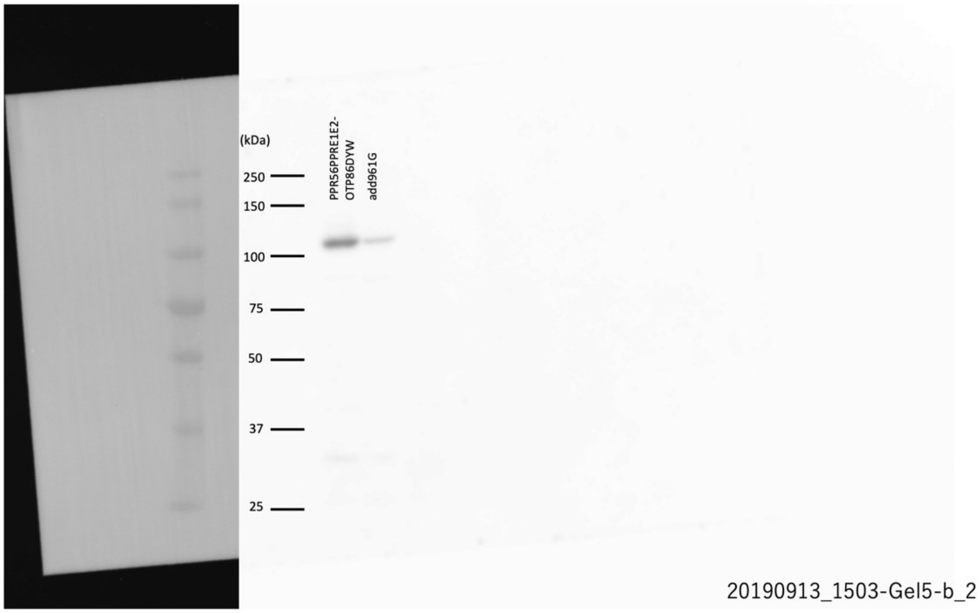
(kDa)
250
150
100
75
50
37
25
PPR56PPRE1E2-OTP86DYW
add961G
20190913_1503-Gel5-b_2

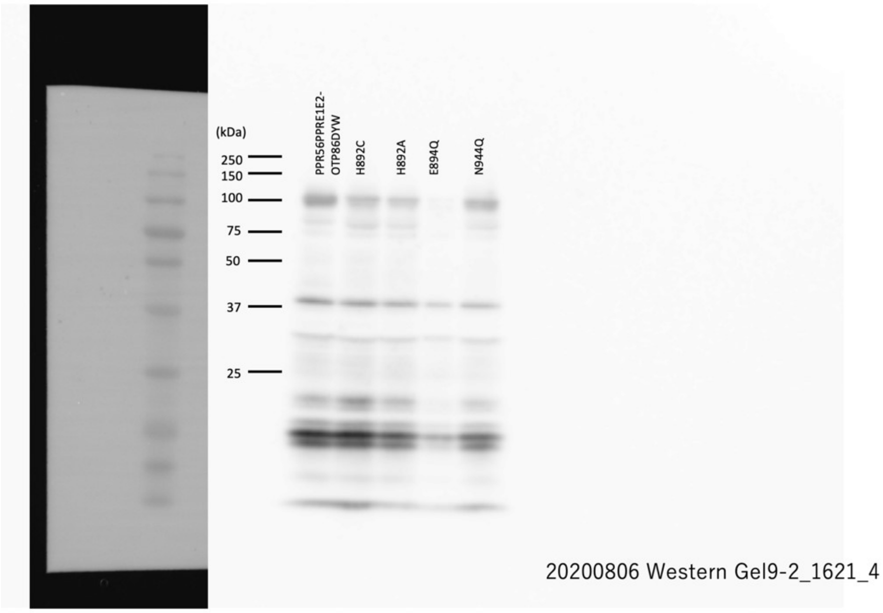
(kDa)
250
150
100
75
50
37
25
PPR56PPRE1E2-OTP86DYW
H892C
H892A
E894Q
N944Q
20200806 Western Gel9-2_1621_4

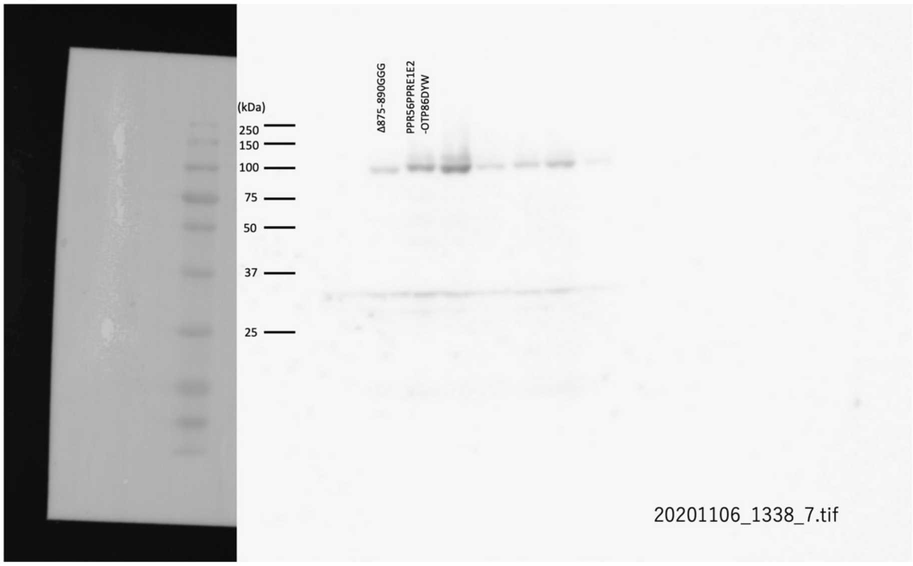

3.) <u>Specificity validation for Anti-6 x His-tag and Anti-Mouse IgG Anti-Mouse IgG.</u>

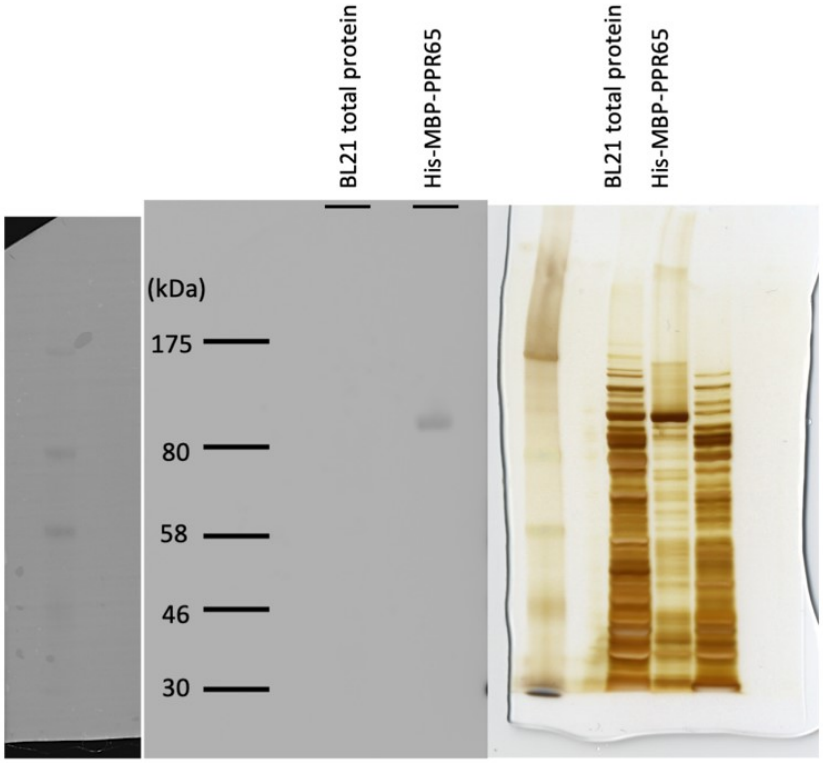

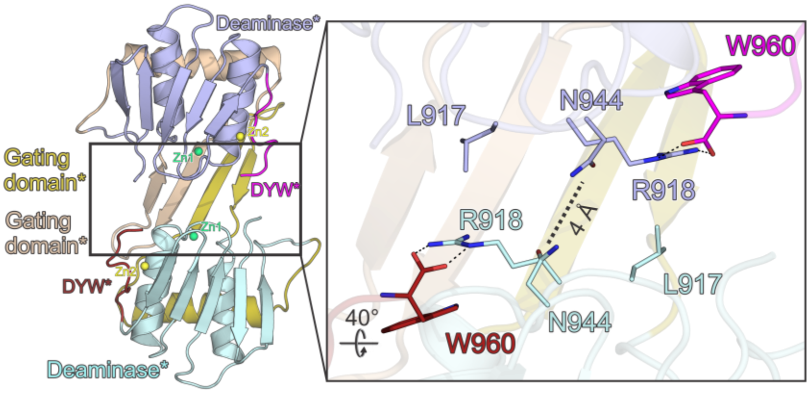

Supplementary Figure 8 Structural implications for DYW* dimerization upon activation. Left – overview of a crystallographic DYW* dimer; Right – close-up of the dimer interface as stick representation. Colours for the first monomer as in Fig. 3c, colours for the second monomer: deaminase domain - grey blue, gating domain - sand, DYW motif - purple. Rotation symbols indicate the views relative to the overview on the left. Rotation symbols indicate the views relative to the overview on the left. Interacting residues are shown as sticks and coloured by atom type. Carbon - as for the respective molecule; nitrogen – blue; oxygen – red; sulphur – yellow. Dashed lines represent hydrogen bonds. Zn1 -green, Zn2 – yellow.

Supplementary Table 1

Data Collection[(a)]	$OTP86^{DYW}$ (inactive)	$OTP86^{DYW*}$ (activated)
Wavelength (Å)	1.282	0.9184
Temperature (K)	100	100
Space Group	C2	$P2_12_12$
Unit Cell (Å, °)	124.568 30.578 77.533<br>90.000 125.748 90.000	117.602 132.887 30.634<br>90.000 90.000 90.000
Resolution (Å)	80 - 2.5 (2.60 - 2.50)	50 - 1.65 (1.75 - 1.65)
Unique Reflections	16019* (1761*)	57997 (8940)
Completeness (%)	99.2 (99.0)	97.7 (94.9)
Redundancy	6.5 (6.0)	12.7 (11.7)
I/σ(I)	7.46 (1.06)	10.32 (1.31)
$R_{sym}(I)$[(b)]	0.21 (1.73)	0.15 (1.72)
CC(1/2)[(c)]	99.4 (42.8)	100.0 (76.4)
**Phasing[(d)]**		
Resolution (Å)	38 – 3.5	-
Zn-sites	4	-
Figure of merit (FOM)	0.27	-
FOM after density modification	0.71	-
**Refinement**		-
Resolution (Å)	38.7 - 2.5	44.0 – 1.65
Reflections		
Number	8482	57767
Completeness (%)	99.53	97.31
Test Set (%)	5.0	3.6
R_{work} / R_{free}[(e)]	0.2215 / 0.2714	0.2208 / 0.2677
ESU (Å)[(f)]	0.41	0.2
Contents of asymmetric unit		
Protein (Molecules)	2	4
Protein (Residues)	257	543
Water Oxygens	33	375
Mean B-Factors ($Å^2$)		
Wilson	43.73	27.93
Protein	47.71	38.91
Water	41.69	34.68
Ramachandran Plot[(g)]		
Favored (%)	95.92	97.01
Outliers (%)	0	0
R.m.s.d.[(h)]		
Bond Lengths (Å)	0.003	0.012
Bond Angles (°)	0.545	1.212
Dihedral Angles (°)	16.64	25.76
Protein Data Bank code	7O4E	7O4F

(a) highest resolution shell in parentheses
(b) $R_{sym}(I) = \Sigma_{hkl}\Sigma_i |I_i(hkl) - \langle I(hkl)\rangle| / \Sigma_{hkl}\Sigma_i |I_i(hkl)|$; for n independent reflections and i observations of a given reflection; $\langle I(hkl)\rangle$ – average intensity of the i observations
(c) Correlation factor CC(1/2) between random half-datasets for reporting results in XDS CORRECT and XSCALE[12]
(d) PHENIX.AUTOSOL [13]
(e) $R = \Sigma_{hkl} ||F_{obs}| - |F_{calc}|| / \Sigma_{hkl} |F_{obs}|$; R_{work} – hkl $\notin$ T; R_{free} – hkl $\in$ T; R_{all} – all reflections;
(f) ESU – estimated overall coordinate error based on maximum likelihood
(h) Calculated with PHENIX.REFINE[14]
(i) R.m.s.d. – root-mean-square deviation from target geometry
* Anomalous data

Supplementary Table 2

Proteins	Resource	ID
AtOTP86	UniprotKB (https://www.uniprot.org)	Q9M1V3
AtDYW1	UniprotKB (https://www.uniprot.org)	P0C7R1
AtDYW2	UniprotKB (https://www.uniprot.org)	Q069S7
PpPPR56	UniprotKB (https://www.uniprot.org)	E0D4J5
PpPPR65	UniprotKB (https://www.uniprot.org)	T2HWD3
AtMEF1	UniprotKB (https://www.uniprot.org)	Q9LTF4
AtMEF8	UniprotKB (https://www.uniprot.org)	Q680H3
AaBo228	https://www.hornworts.uzh.ch	AagrBONN_evm.model.Sc2ySwM_228.5646.1
AaBo368	https://www.hornworts.uzh.ch	AagrBONN_evm.model.Sc2ySwM_368.2386.1
EcCD	PDB (https://www.rcsb.org)	1AF2
MmCD	PDB (https://www.rcsb.org	2FR6

Supplementary Note 1

The proton transfer is detailed in a study about cytidine deaminase mechanism of action[15]. After nucleophilic attack of the hydrolytic water on the C4 of the cytidine, the carboxylic acid of E894 protonates the amino group. The leaving ammonia is stabilized by the carboxylate. There are no further hydrogen bond acceptors in the vicinity, rather a hydrophobic neighbourhood, so the ammonia can diffuse into the nearby bulk solvent.

Supplementary Note 2

Alanine mutants of neither the serine residues 828 and 839 nor K915 have a significant effect on activity which is not surprising since they are likely fostering $OTP86^{DYW}$ inhibition (Fig. 4b, c). A minor loss of activity due to the K915A mutation may be attributed to intrinsic protein instability. We were asking whether K915, S828 and S893 mutually depend on each other and tested the activity of combined mutations. The double mutants S828A/S893A and S828A/K915A show no significant change in activity, while the S893A/K915A and S893A/S828A/K915A mutants have a drastically reduced activity. We reason that the interplay of K915 and S893 during $OTP86^{DYW}$ activation is crucial for either structural stability or catalysis.

Supplementary Note 3

While the negative effect of the N916A mutation may rely on a structural destabilization of the nucleotide pocket, L917 and R918 are part of a potential dimerization interface of the DYW domain which is only observed in the activated $OTP86^{DYW}$* (Supplementary Fig. 8). The interface involves also the β-fingers of the two neighbouring DYW domains, forming a small β-sheet that may consolidate the dimer and thus the active conformation. Lastly, W960 likely plays an important role in the conjectural dimer interface since it stabilizes R918 with both oxygens of its carboxy terminus. This may also explain the missing activities of an add961G mutant, where an additional glycine was added to the proteins C-terminus which likely abolishes the stabilization of R918. A multimerization of PPR65, which shows in vitro deaminase activity, as well as PPR56, has been reported - in coherence with DYW dimerization observed in this study[16]. Interestingly, $OTP86^{DYW}$ N944 also takes part in the dimer interface, forming a weak hydrogen bonding interaction. In *P. patens* PPR65, a glutamine at the equivalent position - and concomitantly stronger

hydrogen bonds - may be responsible for the consolidation of dimerization which likely has allowed the initial description of RNA editing activity in an orthogonal *E. coli* system in this case[17]. Apart from its potential role in dimerization, L917 may also have a base-stacking function during substrate binding which explains the reduced activity of the L917A mutant (see Fig. 4b, c, d, g).

Supplementary Note 4

The DYW domain is likely to fold back to the N-terminus considering the predicted direction and path of the RNA extrapolated from comparison to known cytidine deaminase co-structures (Fig. 4, 7). Therefore, the gating domain is most likely located around the E1 and E2 domains, of which the actual function is so far not clear. Binding of the PPR tract to the target RNA may induce the conformational change of the E1 and/or E2 domain, which may lead to a deaminase activation. This scenario is consistent with the less conserved E1 and E2 domain in DYW1 and DYW2, which only function as a complex with PPR proteins harbouring entire E1 and E2 domains[18–20]. The suppressed deaminase activity of DYW1 and DYW2 monomer is released only upon interaction with the E1 and E2 domains in the partner PPR proteins associated with target RNA.

Supplementary Note 5

Several prior studies support a multimerization of PPR proteins with DYW domains consistent with the conjectural dimerization of DYW domains observed in our work. A multimerization of recombinant PPR65, which deaminates target cytidines in vitro as well as PPR56, was reported[22]. DYW2, which complements the missing C-terminal part of the DYW domain of E+ type PPR proteins, forms a homodimer in plastids[15,17].

Supplementary Note 6

Respective PPR proteins predicted to catalyse the reverse U to C RNA editing process were coined DYW:KP since they harbour a special variant of the DYW motif, composed of DRH, DXX or GRP at the very C-terminus [2,11]. These variants would still support Zn2 coordination within the DYW motif and also dimerization, but may have a different architecture of the neighbouring Zn1 active site region. Alternatively, the unique triplets might be essential to associate with other editosomal proteins in case the DYW motif

recruits other factors. Both OTP86 residues, S828 and S893, which contact K915 and may co-regulate the deamination in OTP86^{DYW}, are alanine in DYW:KP proteins (Fig. 2)[2,11]. Also, the conserved HP motif in the N-terminus of the gating domain, preceding α-helix 1 is missing in more than half of the DYW:KP proteins which may influence the positioning of the respective gating domains (Supplementary Fig. 4). These observations may indicate that U to C amination by DYW:KP does not require strong autoinhibition probably due to other regulation mechanisms, e.g., association with other proteins or amino group donors.

Supplementary References

1. Mueller, U. *et al.* Facilities for macromolecular crystallography at the Helmholtz-Zentrum Berlin. *J. Synchrotron Radiat.* **19**, 442–9 (2012).
2. Gutmann, B. *et al.* The Expansion and Diversification of Pentatricopeptide Repeat RNA-Editing Factors in Plants. *Mol. Plant* **13**, 215–230 (2020).
3. Rüdinger, M., Fritz-Laylin, L., Polsakiewicz, M. & Knoop, V. Plant-type mitochondrial RNA editing in the protist Naegleria gruberi. *RNA* **17**, 2058–2062 (2011).
4. Schallenberg-Rüdinger, M., Lenz, H., Polsakiewicz, M., Gott, J. M. & Knoop, V. A survey of PPR proteins identifies DYW domains like those of land plant RNA editing factors in diverse eukaryotes. *RNA Biol.* **10**, 1549–1556 (2013).
5. Fu, C. J., Sheikh, S., Miao, W., Andersson, S. G. E. & Baldauf, S. L. Missing genes, multiple ORFs, and C-to-U type RNA editing in Acrasis kona (Heterolobosea, Excavata) mitochondrial DNA. *Genome Biol. Evol.* **6**, 2240–2257 (2014).
6. Kumar, S., Stecher, G. & Tamura, K. MEGA7: Molecular Evolutionary Genetics Analysis Version 7.0 for Bigger Datasets. *Mol. Biol. Evol.* **33**, 1870–1874 (2016).
7. Edgar, R. C. MUSCLE: Multiple sequence alignment with high accuracy and high throughput. *Nucleic Acids Res.* **32**, 1792–1797 (2004).
8. Katoh, K., Rozewicki, J. & Yamada, K. D. MAFFT online service: Multiple sequence alignment, interactive sequence choice and visualization. *Brief. Bioinform.* **20**, 1160–1166 (2018).
9. Cheng, S. *et al.* Redefining the structural motifs that determine RNA binding and RNA editing by pentatricopeptide repeat proteins in land plants. *Plant J.* **85**, 532–547 (2016).
10. Wheeler, T. J., Clements, J. & Finn, R. D. Skylign: A tool for creating informative, interactive logos representing sequence alignments and profile hidden Markov models. *BMC Bioinformatics* **15**, 7 (2014).
11. Gerke, P. *et al.* Towards a plant model for enigmatic U-to-C RNA editing: the organelle genomes, transcriptomes, editomes and candidate RNA editing factors in the hornwort *Anthoceros agrestis*. *New Phytol.* **225**, 1974–1992 (2020).
12. Karplus, P. A. & Diederichs, K. Linking Crystallographic Model and Data Quality. *Science* **336**, 1030–1033 (2012).

13. Zwart, P. H. *et al.* Automated Structure Solution with the PHENIX Suite. *Methods Mol. Biol.* **426**, 419–435 (2008).
14. Afonine, P. V. *et al.* Towards automated crystallographic structure refinement with phenix.refine. *Acta Crystallogr. Sect. D Biol. Crystallogr.* **68**, 352–367 (2012).
15. Kazemi, M., Himo, F. & Åqvist, J. Enzyme catalysis by entropy without Circe effect. *Proc. Natl. Acad. Sci. U. S. A.* **113**, 2406–2411 (2016).
16. Hayes, M. L. & Santibanez, P. I. A plant pentatricopeptide repeat protein with a DYW-deaminase domain is sufficient for catalyzing C-to-U RNA editing in vitro. *J. Biol. Chem.* **295**, 3497–3505 (2020).
17. Oldenkott, B., Yang, Y., Lesch, E., Knoop, V. & Schallenberg-Rüdinger, M. Plant-type pentatricopeptide repeat proteins with a DYW domain drive C-to-U RNA editing in Escherichia coli. *Commun. Biol.* **2**, 1-8 (2019).
18. Boussardon, C. *et al.* Two interacting proteins are necessary for the editing of the NdhD-1 site in Arabidopsis plastids. *Plant Cell* **24**, 3684–94 (2012).
19. Andrés-Colás, N. *et al.* Multiple PPR protein interactions are involved in the RNA editing system in Arabidopsis mitochondria and plastids. *Proc. Natl. Acad. Sci.* **114**, 201705815 (2017).
20. Guillaumot, D. *et al.* Two interacting PPR proteins are major Arabidopsis editing factors in plastid and mitochondria. *Proc. Natl. Acad. Sci.* **114**, 201705780 (2017).
